# Phase 1 Study of a Combination AMA1 Blood Stage Malaria Vaccine in Malian Children

**DOI:** 10.1371/journal.pone.0001563

**Published:** 2008-02-13

**Authors:** Alassane Dicko, Issaka Sagara, Ruth D. Ellis, Kazutoyo Miura, Ousmane Guindo, Beh Kamate, Moussa Sogoba, Mohamed Balla Niambelé, Mady Sissoko, Mounirou Baby, Amagana Dolo, Gregory E. D. Mullen, Michael P. Fay, Mark Pierce, Dapa A. Diallo, Allan Saul, Louis H. Miller, Ogobara K. Doumbo

**Affiliations:** 1 Malaria Research and Training Center, Department of Epidemiology of Parasitic Diseases, Faculty of Medicine, Pharmacy and Dentistry, University of Bamako, Bamako, Mali; 2 Malaria Vaccine Development Branch, National Institute of Allergy and Infectious Diseases, National Institutes of Health, Bethesda, Maryland, United States of America; 3 Biostatistics Research Branch, National Institute of Allergy and Infectious Diseases, National Institutes of Health, Bethesda, Maryland, United States of America; London School of Hygiene & Tropical Medicine, United Kingdom

## Abstract

**Background:**

Apical Membrane Antigen-1 (AMA1) is one of the leading blood stage malaria vaccine candidates. AMA1-C1/Alhydrogel® consists of an equal mixture of recombinant AMA1 from FVO and 3D7 clones of *P. falciparum,* adsorbed onto Alhydrogel®. A Phase 1 study in semi-immune adults in Mali showed that the vaccine was safe and immunogenic, with higher antibody responses in those who received the 80 µg dose. The aim of this study was to assess the safety and immunogenicity of this vaccine in young children in a malaria endemic area.

**Design:**

This was a Phase 1 dose escalating study in 36 healthy children aged 2–3 years started in March 2006 in Donéguébougou, Mali. Eighteen children in the first cohort were randomized 2∶1 to receive either 20 µg AMA1-C1/Alhydrogel® or *Haemophilus influenzae* type b Hiberix® vaccine. Two weeks later 18 children in the second cohort were randomized 2∶1 to receive either 80 µg AMA1-C1/Alhydrogel® or *Haemophilus influenzae* type b Hiberix® vaccine. Vaccinations were administered on Days 0 and 28 and participants were examined on Days 1, 2, 3, 7, and 14 after vaccination and then about every two months. Results to Day 154 are reported in this manuscript.

**Results:**

Of 36 volunteers enrolled, 33 received both vaccinations. There were 9 adverse events related to the vaccination in subjects who received AMA1-C1 vaccine and 7 in those who received Hiberix®. All were mild to moderate. No vaccine-related serious or grade 3 adverse events were observed. There was no increase in adverse events with increasing dose of vaccine or number of immunizations. In subjects who received the test vaccine, antibodies to AMA1 increased on Day 14 and peaked at Day 42, with changes from baseline significantly different from subjects who received control vaccine.

**Conclusion:**

AMA-C1 vaccine is well tolerated and immunogenic in children in this endemic area although the antibody response was short lived.

**Trial Registration:**

Clinicaltrials.gov NCT00341250

## Introduction

Malaria remains the primary cause of death in children in sub-Saharan Africa despite the existence of tools such as antimalarial drugs, insecticide treated bet nets, and indoor residual spraying. It is estimated that malaria causes between 300 and 500 million clinical cases and 700,000 to 1.6 million deaths each year [1]. An effective malaria vaccine is a much needed tool to combat this disease. With the international effort to develop a malaria vaccine, several malaria vaccine candidates have reached the stage of clinical testing in malaria exposed populations. Apical membrane antigen-1 (AMA1) is a surface protein expressed during the asexual blood stage of *P. falciparum,* and is a leading vaccine candidate, with several formulations being tested in malaria endemic areas in Africa [2], [3]. Preclinical studies have shown that vaccination with AMA1 induces antibodies and protection against homologous parasite challenge in both rodent and monkey models of malaria infection [4], [5], [6], [7], [8].

The AMA1-Combination 1 (C1) vaccine used in this study contains an equal mixture of the correctly folded ectodomain portion of recombinant AMA1 from two different clones of *P. falciparum*, FVO and 3D7. The combination vaccine was chosen because of sequence polymorphism of the AMA1 gene and the strain specific antibody response to recombinant AMA1 [9], [10]. It is hoped that the inclusion of more than one polymorphic protein will induce broader protection against infection with diverse strains of parasites. Phase 1 studies in malaria naïve adults in the US and in semi-immune adults in Mali have shown that the vaccine is safe and immunogenic at a dose of 80 µg [11], [12]. Based on these results, a Phase 1 clinical trial of this vaccine in children was initiated in a malaria-endemic setting in Donéguébougou in Mali, West Africa.

## Methods

This was a dose-escalating randomized double blind clinical trial designed to assess the safety and immunogenicity of the blood stage malaria vaccine candidate AMA1-C1, adjuvanted with Alhydrogel®. The protocol for this trial and supporting CONSORT checklist are available as supporting information; see Checklist S1 and Protocol S1.

### Participants

Participants were healthy children available for the duration of the trial (52 weeks) aged 2 to 3 years old living in the village of Donéguébougou, a village with high seasonal malaria transmission occurring from July to November [13]. Exclusion criteria included recent or current participation in another investigational vaccine or drug trial, history of severe allergic reaction or asthma, known immunodeficiency, recent use of systemic corticosteroids or immunosuppressive drugs, recent receipt of a licensed vaccine or blood transfusion, history of splenectomy, previous receipt of an investigational malaria vaccine or *Hemophilus influenza B* vaccine, known thrombocytopenia or bleeding disorder, abnormal screening laboratories (alanine aminotransferase (ALT) >62 U/L, serum creatinine >61 mmol/L, hemoglobin <8.5 g/dL, absolute leukocyte count <3000/mm^3^ or >14,500/mm^3^, absolute lymphocyte count <1000/mm^3^, or platelet count <120,000/mm^3^), and any other clinically significant disease or condition which might confound the interpretation of study results.

### Ethics

Community consent was obtained at a meeting with village leaders, heads of families, and other community members prior to the start of the study. Individual informed consent was then obtained after oral translation of the consent form into the local language. Understanding of the contents of the consent was confirmed by means of a multiple choice questionnaire. Parents or guardians unable to read placed an imprint of his/her finger in place of a signature; an independent witness also signed all consent forms. The study was conducted under a protocol reviewed and approved by the Institutional Review Board of the National Institute of Allergy and Infectious Disease (NIAID), and by the Ethics Committee of the Faculty of Medicine, Pharmacy and Dentistry, University of Bamako. The study was submitted to the U.S. Food and Drug Administration for review as part of Investigational New Drug application BB-IND#10944. The study was monitored for regulatory compliance and data quality by the Regulatory Compliance and Human Subjects Protection Branch of NIAID and the Initiative for Vaccine Research of WHO. The NIAID Data Safety Monitoring Board (DSMB) and /or local medical monitor reviewed safety data prior to next vaccination and dose escalation.

### Interventions

Both recombinant cGMP AMA1-FVO and AMA1-3D7 are 62 kDa proteins consisting of amino acids 25 through 545 of the published sequences of each line's AMA1 gene (GenBank accession number AJ277646 for FVO and accession number U65407 for 3D7). Protein production and vaccine formulation are described in detail elsewhere [7]. AMA1-C1/Alhydrogel® malaria vaccine was supplied as a slightly turbid suspension in single-dose vials. Each 2.0 mL vial contains a single dose, of which 0.5 mL is the intended volume to be injected. Half mL of vaccine contains about 424 µg of aluminum (Alhydrogel®, HCI Biosector, Denmark) per dose onto which either 20 µg or 80 µg of recombinant AMA1-C1 has been bound. The product conforms to established requirements for endotoxin, sterility, and general safety. The AMA1-C1/Alhydrogel® research products for this protocol were supplied by the Pharmaceutical Development Section, Pharmacy Department, Clinical Center of National Institutes of Health, where the AMA1-C1/Alhydrogel® vaccine was formulated and vialed. Both the AMA1-C1/Alhydrogel® and Hiberix® (GlaxoSmithKline, Uxbridge, UK) vaccines were transported and stored at 0.5°C to 9°C; temperature recording devices accompanied the vaccines at all times to ensure storage temperature limits were not violated.

Hiberix® is a noninfectious vaccine containing purified capsular polysaccharide of *Haemophilus influenzae* type b (Hib) covalently bound to tetanus toxoid. Hiberix® is supplied as a white lyophilized pellet for reconstitution with sterile saline solution 0.9%. Each 0.5 mL dose contains 10 µg of purified polysaccharide covalently bound to approximately 30 µg of tetanus toxoid. Hiberix® meets the World Health Organization requirements for the manufacture of biological substances and Hib conjugate vaccines. Both vaccines were administered IM in the thigh muscles on Days 0 and 28, in alternating legs.

### Outcomes: Safety and Tolerability

Local and systemic adverse events were recorded and graded by severity and relationship to vaccine. Volunteers remained in the study clinic for at least 30 minutes after each immunization to evaluate for immediate adverse reactions. Follow up visits were scheduled for Days 1, 2, 3, 7, and 14 after each vaccination, and at study weeks 12, 22, 30, 42, and 52. Solicited injection site reactions were pain, erythema, and swelling; solicited general adverse events were fever, headache, nausea, malaise, myalgia, arthralgia, urticaria, drowsiness, irritability, and loss of appetite. Investigators also recorded any other adverse events which occurred during the follow up period. Injection site erythema, swelling, and induration were graded as follows: 0 = absent, 1 = 0–20 mm, 2 = >20–50 mm, 3 = >50 mm. Fever (axillary) was graded as 0 = <37.5°C, 1 = 37.6–38°C, 2 = >38–39°C, and 3 = >39°C. Pain and solicited adverse events other than fever and urticaria were graded as follows: 0 = absent/none, 1 = easily tolerated, 2 = interferes with daily activity, 3 = prevents daily activity. Non-solicited adverse events were graded as 0 = none, 1 = no effect on activities of daily living, 2 = partial limitation in activities of daily living, or minimal intervention required, 3 = activities of daily living limited to <50% of baseline, or medical evaluation with intervention required. Hematological (Hb, WBC, and platelets) and biochemical (ALT, and creatinine), laboratory parameters were measured at screening, on days of immunization, and on Days 3 and 14 after each vaccination. Hematology was also performed on Day 7 after each vaccination and at weeks 12, 22, 30, 42, and 52. Serious adverse events (SAEs) were defined as any adverse event resulting in death, life threatening, requiring hospitalization, resulting in disability or incapacity, or any other event which required intervention to prevent such outcomes.

### Outcomes: Immunogenicity

Antibody responses to the AMA1 antigens were measured in plasma by enzyme linked immunosorbent assay (ELISA) at Days 0, 14, 42, 98 and 154. The ELISA technique used was described previously [11]. A human anti-AMA1 standard plasma was made using a pool of plasma from 3 individuals receiving AMA1-C1 immunization in a US vaccine trial (manuscript in preparation). The standard pool was assigned 460.9 ELISA units on AMA1-FVO and 578.0 units on AMA1-3D7.The minimal detection level of this assay was 28 ELISA units and all data less than 28 ELISA units were assigned a value of 14 units for statistical analysis.

### Sample size

Sample size for the Phase 1 part of the study was based on safety outcomes. Group sizes of 10 provide an 80% likelihood of detecting adverse events that occur with a frequency of 15%. An extra two subjects per group were included in case of loss to follow up.

### Randomization

Participants in the two treatment cohorts (20 µg AMA1-C1/Alhydrogel and 80 µg AMA1-C1/Alhydrogel) were randomized in a 2∶1 ratio to receive either the study vaccine or the control (Hiberix®). Randomization codes were created by the study statistician, and randomization occurred at the time of first vaccination. A copy of the randomization code was provided to the pharmacist who used coded labels for the vaccines, and to the medical monitor and DSMB.

### Blinding

The study was double blinded, with both participants and investigators unaware of treatment assignment until completion of the study. Since the appearance of the vaccines was slightly different, opaque tape was placed over the syringe so that investigators were unable to see the contents. Unblinding of the investigators occurred after adverse event and immunologic databases to Day 154 were cleaned and finalized.

### Statistical methods

Exact Jonckheere–Terpstra (J-T) tests were performed to examine dose-response relationships for adverse events, according to dose of AMA1-C1 vaccine (control, low dose, and high dose) for each category of AE and total number of AEs. For each AE category tested, each subject was defined as having a response of no AE, grade 1 AE, grade 2 AE, grade 3 AE or SAE based on the subjects highest grade of AE in that category. In order to maintain a low threshold for detecting safety concerns no correction was made for multiple comparisons. For each subject, the arithmetic average of the FVO and 3D7 ELISA responses at each day was used as that subject's AMA1-C1 antibody response for that day. The primary immunological outcome in this trial was the difference in AMA1-C1 antibody response between Day 42 and Day 0. Dose response was tested by exact J-T test. Similar J-T tests were performed for other vaccine days using the differences in antibody response between other time points (Days 14, 98 and 154) and Day 0. All ELISA data for two subjects were excluded from the antibody response analyses, because there appeared to be specimen mishandling in those two cases. The decision to exclude these subjects was made before unblinding. No other subjects were excluded from the immunogenicity analysis, and those two subjects were included in all safety analyses. The concordance between log transformed antibody levels against AMA1-FVO and AMA1-3D7 was measured with random marginal agreement coefficients with squared difference as suggested in Fay (2005) [14].

## Results

### Participant Flow and Baseline Data

**Figure 1:**
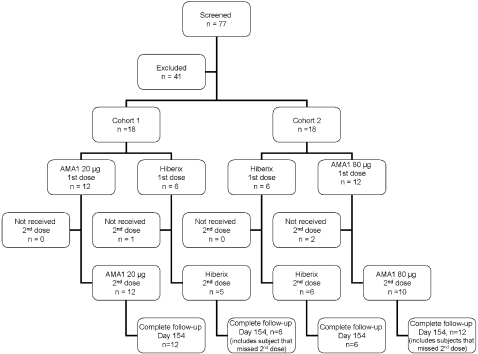
CONSORT Flow Chart.

Seventy seven children were screened for inclusion in the study, of whom 36 (19 males and 17 females) were enrolled (Figure 1). Reasons for exclusion were concurrent illness (n = 13), positive serology for chronic hepatitis B or C (n = 8), hemoglobin <8.5 g/dL and/or malnutrition (n = 6), other abnormal screening laboratory tests (n = 7), age outside the specified range (n = 6), and one subject was eligible but not enrolled as enrollment was complete. The first cohort (12 subjects receiving 20 µg AMA1-C1/Alhydrogel and 6 subjects receiving Hiberix®) was vaccinated on March 16, 2006 and the second cohort (12 subjects receiving 80 µg AMA1-C1/Alhydrogel and 6 receiving Hiberix®) was vaccinated about 2 weeks later. The second vaccination of the 2^nd^ cohort was completed about 2 months prior to the start of malaria transmission season. Three subjects (1 in Hiberix® group and 2 in the 80 µg dose group) did not receive the second vaccination due to acute Hepatitis A infection. All the participants completed followed up to Day 154.

### Safety

Both doses of the AMA1-C1 vaccine were well tolerated (Table 1). A total of 9 adverse events that were considered definitely, probably or possibly related to the vaccination occurred in 7 subjects who received the AMA1-C1 vaccine. Most (5/9) were local injection site reactions. All were mild to moderate in severity and resolved within 4 days except one case of elevated ALT that persisted through the end of the follow up period. This subject was enrolled with ALT of 47.3 U/L (near the upper limit of normal range 49.6 U/L). His ALT increased to 72.8 U/L at Day 3, decreased to 59.4 U/L on Day 14 but remained slightly above the normal range throughout the follow up period. Tests for hepatitis A, B and C in this individual were repeatedly negative. As no apparent etiology was found it was classified as possibly related to the vaccination. There was no significant relationship between AMA1-C1 dose and either severity of AE overall or in any specific category of AEs. There were no severe or serious adverse event deemed related to the vaccination in any group. Three serious adverse events (one in each group) were reported. All were related to acute viral Hepatitis A (two hospitalizations and one Grade 4 elevation of ALT protocol defined as an SAE) and were considered unrelated to the vaccination.

**Table 1:** Local injection site, systemic and laboratory adverse events judged definitely, probably, or possibly related to vaccine after vaccination with the AMA1-C1/Alhydrogel® or Hiberix® vaccines.

	Vaccination #1			Vaccination #2			Vaccinations #1 and #2		
	AMA1-C1		Hiberix® (n = 12)	AMA1-C1		Hiberix® (n = 11)	AMA1-C1		Hiberix®
	20 µg (n = 12)	80 µg (n = 12)		20 µg (n = 12)	80 µg (n = 10)		20 µg	80 µg	
**Local**									
Pain	1	1	1	0	1	0	1	2	1
Swelling	1	0	0	1	0	0	2	0	0
Erythema	1	0	1	0	0	0	1	0	1
**Systemic**									
Fever	0	0	1	1	0	0	1	0	1
Vomiting	0	0	1	0	0	0	0	0	1
Anorexia	0	0	1	0	0	0	0	0	1
Drowsiness	0	0	1	0	0	0	0	0	1
**Laboratory**									
Elevated WBC	1	0	0	0	0	1	1	0	1
Elevated ALT	1	0	0	0	0	0	1	0	0

### IgG responses to AMA1-3D7 and AMA1-FVO

Antibody levels against AMA1-3D7 and AMA1-FVO were measured by ELISA at Day 0 prior to vaccination, Days 14, 42, 98 and 154. Data for two subjects (one in the Hiberix® group and another in the 80 µg AMA1-C1 dose group) were excluded because of a labeling error on study Day 42. Of the 34 subjects considered for the immunology analysis, pre-existing antibodies to AMA1-FVO and AMA1-3D7 were detectable in 14 (5 in the Hiberix® group, 4 in the 20 µg dose group and 5 in the 80 µg dose group) and 17 (7 in the Hiberix® group, 4 in the 20 µg dose group and 6 in the 80 µg dose group) respectively. Three subjects (two in the Hiberix® group and one in the 20 µg dose group) had high antibody levels (greater than 1000 units) prior to vaccination.

Individual antibody values for the FVO and 3D7 antigens are shown in Table 2. Using all 34 subjects, there was high concordance between the log transformed antibody response to AMA1 FVO and AMA1 3D7 both before [0.92 with 95% C.I. (0.85, 0.96) at Day 0] and after vaccinations [0.96 with 95% C.I. (0.93, 0.98) at Day 42].

**Table 2:** ELISA antibody values in plasma from volunteers on selected time points.

		AMA1-FVO					AMA1-3D7				
Subject no	Vaccine	Day 0	Day 14	Day 42	Day 98	Day 154	Day 0	Day 14	Day 42	Day 98	Day 154
1*	Hiberix	14	14	63	14	35	32	14	40	14	73
6		14	14	14	14	14	14	14	14	14	14
8		14	14	14	14	14	14	14	14	14	14
10		14	14	14	14	14	14	14	14	14	14
13		14	14	14	14	70	14	14	14	14	157
14		33	33	14	14	14	58	47	14	14	14
25		365	263	84	14	14	305	215	78	14	14
28		14	14	14	14	85	36	29	14	14	80
30		257	249	199	450	167	123	115	88	175	74
32		1573	1175	663	362	419	2386	2340	1110	605	613
33		2416	1928	1175	471	451	2608	2084	1175	453	642
2	20 µg	14	82	273	48	46	14	127	239	65	52
3		14	53	144	14	113	14	77	187	30	91
4		14	565	927	187	127	14	824	997	193	139
5		28	140	251	81	40	14	131	193	54	14
7		14	127	384	70	35	14	112	413	102	14
9		14	64	397	155	82	14	279	1188	492	252
11		2043	1480	1051	408	274	462	530	687	264	176
12		14	81	627	302	218	28	119	724	249	154
15		14	70	300	219	230	14	121	561	244	200
16		120	681	1427	325	174	52	425	1134	258	148
17		14	14	356	98	32	14	14	225	67	14
18		62	545	471	54	14	77	742	730	104	14
19	80 µg	29	47	114	32	14	73	110	192	56	30
20		14	58	157	37	14	14	67	201	39	14
22*		68	177	144	40	125	50	199	292	91	111
23		31	46	156	79	397	14	34	167	117	1009
24*		14	34	14	14	14	14	14	14	14	14
26		14	245	530	167	93	68	958	1520	453	214
27		14	37	113	14	14	14	143	275	84	32
29		30	85	181	36	78	31	82	224	64	79
31		115	597	2640	454	146	49	268	1258	227	78
34		14	288	746	157	168	14	248	780	228	156
35		14	1983	3944	679	1200	29	2243	4348	674	1053

* did not received the second vaccination due Hepatitis A.

**Figure 2:**
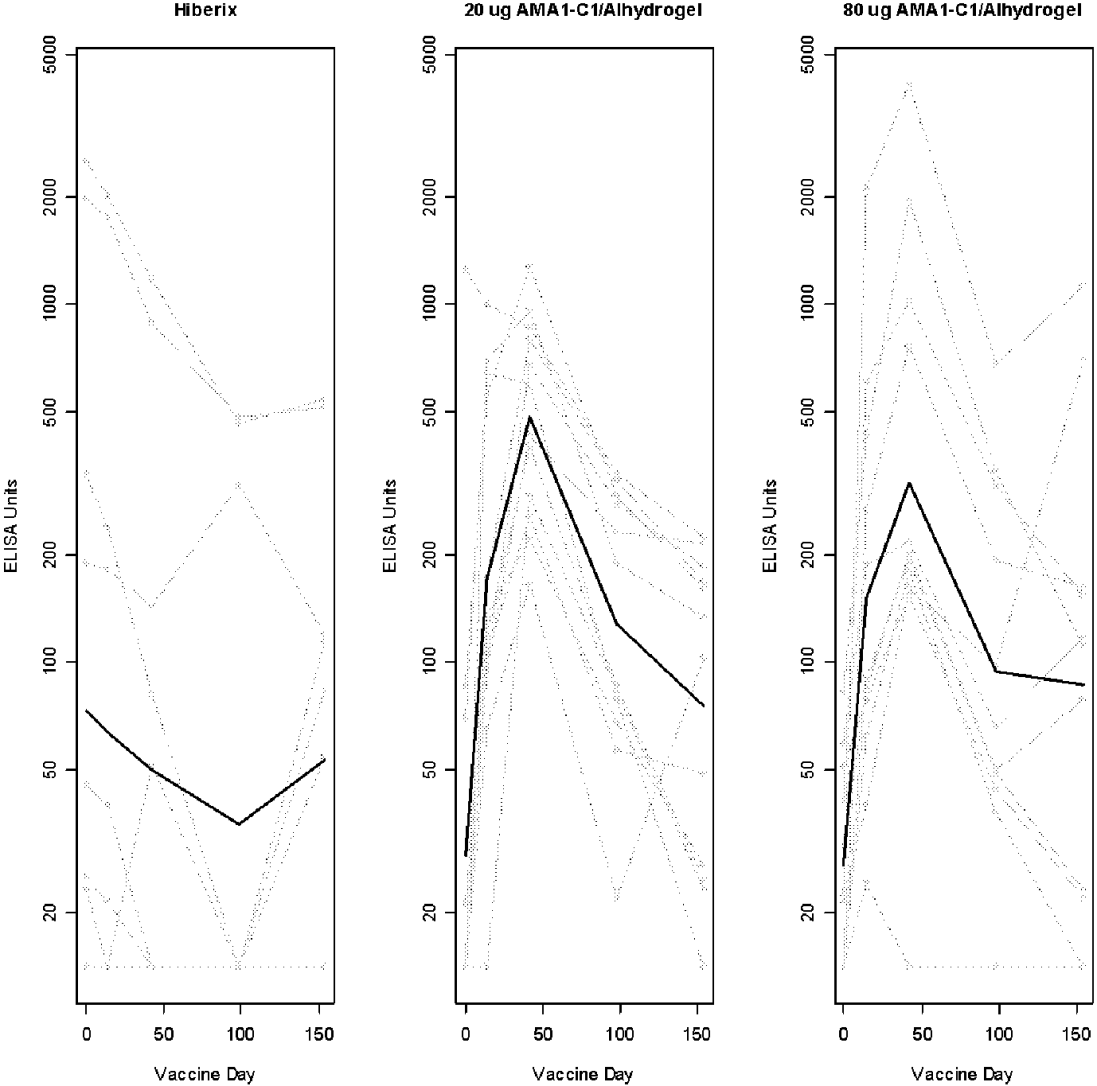
Average of FVO and 3D7 ELISA responses by Vaccine Day. Gray lines are individual subjects, black lines are geometric means within each randomization group.

For the test vaccine, an increase in the average of FVO and 3D7 responses was seen at Day 14 and continued to increase after the second vaccination (Day 42), but decreased by Days 98 and 154 (Figure 2). In the 20 µg group, the geometric mean antibody response (average FVO and 3D7) increased from 29.0 antibody units (95% CI 12–68.3) on Day 0 to 171.0 units (95% CI 76.6–372.3) on Day 14 and to 483 units (95% CI 319.7–729.7) on Day 42. Similarly, in the 80 µg dose group the mean antibody response increased from 27.5 units (95% CI 17.5–42.0) to 151.7 units (95% CI 62.7–367.4) and to 317.9 units (95% CI 113.1–893.9) on Day 42 while the corresponding mean antibody levels in the Hiberix® group were 73.1 units (95% CI 19.0–281.4), 63.8 units (95% CI 17.0–238.8) and 49.8 units (95% CI 15.8–157.3). The dose responses were significant at Days 14 and 42 (p values 0.0003, 0.0008, respectively).

Although there were still statistically significant dose responses at Days 98 (p = 0.0026) and 154 (p = 0.0170), by Day 98 the antibody responses in the 20 µg and 80 µg dose groups decreased to geometric mean levels about 3 times higher than those in subjects who received Hiberix®, with geometric means of 127.3 (95% CI 73.2–221.2) in the 20 µg dose group, 94.0 (95% CI 43.2–205.0) in the 80 µg dose and 35.2 (95% CI 12.2–102.0) in the Hiberix® group. These levels decreased further by Day 154.

## Discussion

### Interpretation

This Phase 1 study of AMA1-C1 adjuvanted with Alhydrogel at the dose of 20 and 80 µg in malaria exposed children in Mali showed that the vaccine was well tolerated, with no vaccine related severe or serious adverse event. This is consistent with the findings in two previous Phase 1 trials in malaria naïve and semi-immune adults [11], [12].

The vaccine induced antibodies to both constituent alleles, with responses to AMA1-FVO and AMA1-3D7 that were highly correlated, as seen in previous studies [11], [12]. Responses were seen 2 weeks after the first vaccination and further increased after the second vaccination. This is different from the study in malaria-naïve US adults [11], where no responses were seen after the first vaccination. This is likely due to prior malaria exposure, as shown by pre-existing antibody in 59% of subjects overall at baseline. Antibody responses after only one dose were also seen in semi-immune Malian adults [12]. However, compared to Malian adults, antibody levels were much lower overall both at baseline and after immunizations (geometric means of 1081.5 and 2634.9 units respectively at baseline and Day 42 in the 80 µg dose adult group) likely due to less prior exposure to malaria in children versus adults at this site [12]. While dose responses were significant at all time points tested, mean antibody levels for the 20 µg and 80 µg dose groups at Days 14 and 42 were similar, as shown by substantially overlapping confidence intervals.

In the US adult study with the same vaccine, as in other malaria vaccine studies in children [15], [16], [17], highest antibody responses were seen after the third dose of vaccine, and it is possible that a third dose is also needed in children in this endemic area, although in the Malian adult study with the same vaccine limited boosting was seen after the third dose [12]. Short half lives of IgG1 and IgG3 antibodies to merozoite antigens in children living in endemic areas have been reported in epidemiological studies in Mali [18] and in Kenya [19]. In the Kenya study the mean half lives of IgG1 and IgG3 in children after severe malaria attack were 9.1 days (95% CI 7.6 and 12.0) and 6.1 days (95% CI 3.7–8.4 days) with little or no boosting after re-infection. Further studies are needed on the mechanisms of the immune response to merozoite antigens as well as the dynamics of these responses and their relationship to malaria infection and disease. Additionally, the ability of vaccination to induce a long term memory response should be further investigated.

### Generalizability

This is the first Phase 1 study of an AMA 1 vaccine in children. It aimed to provide information on the safety and immunogenicity of this vaccine in this population in a malaria endemic area prior to a larger Phase 2b study. The study population was chosen to represent young children living in an area with seasonal intense malaria transmission and the results should be broadly generalizable to such populations. Like any Phase 1 study, it is powered to detect only frequent events or large differences. After reviewing the safety data in this study, a Phase 2 study was initiated. The Phase 2 study will provide further information on the safety and the immunogenicity of the AMA1-C1/Alhydrogel vaccine, as well as the biologic impact on parasitemia during the malaria transmission season. Although there is an increase in antibody in subjects who received the AMA1-C1 vaccine, the level of antibody that would lead to protection is unknown.

### Overall evidence

This trial provided the first data on the safety and immunogenicity of AMA1–C1 vaccine in malaria exposed children. It shows that the vaccine is well tolerated and immunogenic although the duration of the induced antibody is short. The absence of safety concerns has justified a Phase 2b study. In addition to assessing biologic impact, the Phase 2b study will provide more information on both safety and immunogenicity as well as whether or not immunization during the transmission season leads to higher antibody responses to AMA1.

## Supporting Information

Checklist S1 CONSORT Checklist
(0.19 MB PDF)

Protocol S1 Trial Protocol
(0.45 MB PDF)
