# Supplementary material for: Phase 1 Study of a Combination AMA1 Blood Stage Malaria Vaccine in Malian Children
Source: PLoS One. 2008 Feb 13;3(2):e1563. doi: 10.1371/journal.pone.0001563 (PMC2216433; doi:10.1371/journal.pone.0001563)
Supplement: Protocol S1 — Trial Protocol (0.45 MB PDF) [file pone.0001563.s002.pdf]

RANDOMIZED, CONTROLLED, PHASE 1/2 STUDY OF THE SAFETY AND IMMUNOGENICITY OF AMA1-C1/ALHYDROGEL® VACCINE FOR *PLASMODIUM FALCIPARUM* MALARIA IN CHILDREN IN DONÉGUÉBOUGOU AND BANCOUMANA, MALI

Version:	2.0 (January 25th, 2006)
NIAID Protocol Number:	281
FMPOS Protocol Number:	Malaria-004
FDA IND Number	BB-IND #10944
Ionizing Radiation:	None
Multi-institution:	Yes
Project Assurance:	FWA #0001769
Proposed start date:	February 2006
Proposed end date:	August 2007

<u>Sponsored by:</u>
Regulatory Compliance and Human Subjects Protection Branch (RCHSPB)
Office of Clinical Research (OCR), Office of the Director (OD)
National Institute of Allergy and Infectious Diseases (NIAID)
National Institutes of Health (NIH)

Team Roster

**Principal Investigator for Study:**	**Alassane Dicko, MD, MS**<br>*Malaria Research and Training Center (MRTC)*<br>*Department of the Epidemiology of Parasitic Diseases (DEAP)*<br>*Faculty of Medicine, Pharmacy, and Odonto-Stomatology (FMPOS), University of Bamako*<br>*BP 1805, Point G*<br>*Bamako, Mali*<br>*Tel/Fax: 223-222-8109*<br>*Email: adicko@mrtcbko.org*
**Site PI for Donéguébougou**	Alassane Dicko, MD, MS<br>*Malaria Research and Training Center (MRTC)*<br>*Department of the Epidemiology of Parasitic Diseases (DEAP)*<br>*Faculty of Medicine, Pharmacy and Odonto-Stomatology (FMPOS),University of Bamako*<br>*BP 1805, Point G*<br>*Bamako, Mali*<br>*Tel/Fax: 223-222-8109*<br>*Email: adicko@mrtcbko.org*
**Site PI for Bancoumana**	Issaka Sagara, MD, MSPH<br>*Malaria Research and Training Center (MRTC)*<br>*Department of the Epidemiology of Parasitic Diseases (DEAP)*<br>*Faculty of Medicine, Pharmacy and Dentistry (FMPOS), University of Bamako*<br>*BP 1805, Point G*<br>*Bamako, Mali*<br>*Tel/Fax: 223-222-8109*<br>*Email: isagara@mrtcbko.org*
**Co-Principal Investigator:**	Louis H. Miller, MD<br>*Malaria Vaccine Development Branch (MVDB)*<br>*National Institute of Allergy and Infectious Diseases (NIAID)*<br>*5640 Fishers Lane, Twinbrook 1*<br>*Rockville, MD 20852*<br>*(301) 435-2177*<br>*Email:Lmiller@niaid.nih.gov*

Senior Investigators:

Ogobara Doumbo, MD, PhD
MRTC/DEAP/FMPOS
University of Bamako,
Bamako, Mali
Dapa Aly Diallo, MD
MRTC/Dept. of Hematology/FMPOS
University of Bamako
Bamako, Mali

Allan Saul, PhD
Gregory Mullen, PhD
MVDB/NIAID
Rockville, MD

Clinical Investigators:

Mahamadou Aly Thera, MD, MPH
Mohamed B. Niambélé, MD
Moussa Sogoba, MD
Beh Kamate, MD
Mahamadou H. Assadou, MD
Ousmane Guindo, MD
Sory Ibrahim Diawara, MD
Belco Poudiougou, MD, MS
Abdoul Baki I Diallo, MD
Mamadi Kone, MD
Moussa Konaré, MD
Abdoulaye Traoré, MD
MRTC/DEAP/FMPOS
University of Bamako
Bamako, Mali

Consultant:

Christopher V. Plowe, MD, MPH
Center for Vaccine Development
University of Maryland
Baltimore, Maryland

Laboratory Investigators:

Amagana Dolo, PharmD, PhD
Mounirou Baby, PharmD
Mady Sissoko, PharmD
Boubacar Traoré, PharmD, PhD
Daniel Yalcouyé, PharmD
Falaye Keita, PharmD
Abdou Salam Keita, MS
Mamadou Ba, MS
Modibo Daou, PharmD
MRTC/DEAP/FMPOS

University of Bamako
Bamako, Mali

Carole Long, PhD
Andrew Orcutt, MS
Olga Murgatov, PhD
Hong Zhou
Sam Mortez, MS
MVDB/NIAID
Rockville, MD

NIAID Regulatory Affairs:

John Tierney, RN
OCR, OD
NIAID, NIH
6700-B Rockledge Drive, MSC 7609
Bethesda, MD 20892-7609
(301) 451-5136

Medical Monitor:

Dr. Tatiana Keita
Clinique Pasteur,
Bamako, Mali
+223 229 1010

Participating Sites

**Clinical Trial Sites:**	**Donéguébougou Vaccine Center**<br>MRTC/DEAP/FMPOS<br>Donéguébougou, Mali<br>**Bancoumana Vaccine Center**<br>MRTC/DEAP/FMPOS<br>Bancoumana, Mali

Participating Laboratories

**Clinical Laboratory:**	**MRTC/DEAP Clinical Laboratory**<br>FMPOS, University of Bamako<br>BP 1805, Point G<br>Bamako, Mali
**Immunology Laboratories:**	**Malaria Vaccine Development Branch**<br>NIAID/NIH<br>5460 Fishers Lane, Twinbrook I<br>Rockville, MD 20852<br>Tel: (301) 435-3405
	**MRTC/DEAP Immunology Laboratory**<br>FMPOS, University of Bamako,<br>BP 1805, Point G<br>Bamako, Mali

Table of Abbreviations

AE	Adverse Event
AMA1	Apical Membrane Antigen-1
AMA1-C1	Apical Membrane Antigen-1 Combination-1 Vaccine
CRF	Case Report Form
DEAP	Département d'Epidémiologie des Affections Parasitaires (Department of the Epidemiology of Parasitic Diseases)
DSMB	Data Safety Monitoring Board
EIR	Entomologic Inoculation Rate
ELISA	Enzyme linked immunosorbent assay
EPI	Extended Program for Immunization
FDA	Food and Drug Administration
FMPOS	Faculté de Medicine, Pharmacie, et Odonto-Stomatologie (Faculty of Medicine, Pharmacy, and Odonto-Stomatology)
FWA	Federal Wide Assurance
GCP	Good Clinical Practice
GIA	Growth Inhibition Assay
HAART	Highly Active Anti-Retroviral Therapy
HCV	Hepatitis C virus
ICH	International Conference on Harmonization
IRB	Institutional Review Board
MMVDU	Mali Malaria Vaccine Development Unit
MRTC	Malaria Research and Training Center
MVDB	Malaria Vaccine Development Branch
NIAID	National Institute of Allergy and Infectious Diseases
NIH	National Institutes of Health
NMCP	National Malaria Control Program
PI	Principal Investigator
RCHSPB	Regulatory Compliance & Human Subjects Protection Branch
PNLP	Programme National de Lutte contre le Paludisme (National Program to Fight Malaria)
SAE	Serious Adverse Event
WHO	World Health Organization

Table of Contents

Team Roster ... 2
Table of Abbreviations ... 6
Protocol Summary ... 11
1.0 Introduction ... 14
1.1 Background ... 14
1.1.1 Malaria as a Public Health Problem ... 14
1.1.2 Disease Burden in Mali ... 14
1.2 The AMA1 Protein ... 14
1.2.1 AMA1-C1 Vaccine ... 16
1.3 AMA1-C1 Vaccine Description ... 16
1.3.1 AMA1 ... 16
1.3.2 Alhydrogel® ... 17
1.3.3 Vaccine Production & Formulation ... 17
1.4 Clinical Experience with AMA1-C1/Alhydrogel® Vaccine ... 18
1.5 Clinical Development Plan ... 19
1.6 Comparator Vaccine ... 19
1.6.1 Rationale for Use of a Comparator Vaccine ... 19
1.6.2 Rationale for Use of the Haemophilus influenzae type B (Hib) Vaccine as a Comparator ... 19
2.0 Objectives ... 20
2.1 Primary Objective ... 20
2.2 Secondary Objectives ... 20
3.0 Study Sites ... 20
4.0 Study Design ... 21
4.1 Overall Design ... 21
4.2 Sample Size and Estimated Duration of Study ... 23
4.3 Timing of Trial ... 23
4.4 Group Allocation ... 23
4.4.1 Phase 1 Study ... 23
4.4.2 Phase 2 Study ... 24
4.5 Blinding ... 25
5.0 Selection and Enrollment of Volunteers ... 25
5.1 Inclusion Criteria ... 25
5.2 Exclusion Criteria ... 25
5.2.1 Rationale for Use of Clinical Assessments of Immunosuppression ... 26
5.3 Treatments That Could Potentially Interfere with Vaccine-Induced Immunity .. 27
5.4 Contraindications to Vaccination ... 27
5.5 Indications for Deferral of Vaccination ... 27
5.6 Subject Withdrawal Criteria ... 28
6.0 Vaccine Preparation ... 28
6.1 Supply of AMA1-C1/Alhydrogel® ... 28
6.2 Supply of Comparator Vaccine ... 29
6.3 Vaccine Storage ... 29
6.4 Vaccine Accountability ... 29
6.5 Disposition of Used/Unused Supplies ... 29
7.0 Study Procedures ... 30
7.1 Consent Procedures ... 30
7.2 Individual Recruitment and Informed Consent ... 30
7.3 Randomization Process ... 31
7.4 Enrollment ... 32

7.5 Immunization Procedure 32
7.6 Clinical Monitoring and Evaluation 32
7.6.1 Vaccination and Vaccine Follow Up 32
7.6.2 Parasitological Follow Up 35
7.6.3 Disease Follow Up 36
7.7 Treatment for Malaria 36
7.8 Laboratory Testing 36
7.9 Immunologic Testing 37
7.9.1 Antibody Assay 37
7.9.2 Plasmodium falciparum Genotyping 37
7.9.3 Growth Inhibition Assay 37
8.0 Adverse Events Monitoring and Reporting 38
8.1 Definitions 38
8.1.1 Adverse Event 38
8.1.2 Serious Adverse Event (SAE) 38
8.2 Assessment of Adverse Events 38
8.2.1 Identification of AEs 38
8.2.2 Determination of Severity 39
8.2.3 Association with Receipt of the Study Vaccine 39
8.2.4 Clinical Malaria 40
8.3 Adverse Event Reporting 40
8.3.1 Serious Adverse Event Reporting 40
8.3.2 Other Adverse Event Reporting 41
8.4 Adverse Event Monitoring 41
8.4.1 Local Medical Monitor 41
8.4.2 Data and Safety Monitoring Board (DSMB) 41
8.5 Criteria for Placing the Study on Hold 42
8.6 Approval to Continue the Study 43
9.0 Data Collection and Monitoring 44
9.1 Source Documentation 44
9.2 Study Documentation 45
9.2.1 Study Reports 45
9.3 Retention of Records 46
9.4 Protocol Revisions 46
9.5 Clinical Investigator's Brochure 46
9.6 Study Monitoring 46
10.0 Statistical Considerations 47
10.1 General Design 47
10.1.1 Description of the Statistical Methods to be Employed 47
10.1.2 Safety 49
10.1.3 Immunogenicity Analysis 49
10.1.4 Premature Termination 49
10.2 Sample Size 49
11.0 Protection of Human Subjects 51
11.1 Institutional Review Board/Ethics Committee 51
11.2 Informed Consent 51
11.3 Risks 52
11.3.1 Venipuncture 52
11.3.2 Immunization with AMA1-C1/Alhydrogel® 52
11.3.3 Immunization with Comparator Vaccine 52
11.4 Precautions Taken to Minimize Risks 52
11.4.1 Immunization 52

11.4.2 Protection of Study Staff.. 52
11.5 Benefits 53
11.6 Confidentiality 53
11.7 Compensation 54
12.0 References... 55
Appendix A – Malaria Vaccine Consent Comprehension Exam..................................... 57
Appendix B – Schedule of Visits.. 58
Appendix C – Summary of Parasitological Follow Up 59
Appendix D - Table of Assessment of Adverse Event Intensity 60
Appendix E – Toxicity Table for Grading Laboratory Adverse Events 61

List of Tables

Table 1 Summary of AMA1-C1/Alhydrogel® Phase 1 Trials 18
Table 2 Summary of Safety Review Meetings and Vaccination Schedule.................................. 43

STATEMENT OF THE DIRECTOR OF THE MALARIA RESEARCH AND TRAINING CENTER OF THE DEPARTMENT OF THE EPIDEMIOLOGY OF PARASITIC DISEASES, FACULTY OF MEDICINE, PHARMACY, AND DENTISTRY, UNIVERSITY OF BAMAKO, MALI:

I, the undersigned, have reviewed this protocol and have approved it. I will ensure that the clinical study as described will adhere to the principles of the ICH/GCP as well as all applicable regulatory requirements. I have read and understood the contents of the Investigator's Brochure provided by the Malaria Vaccine Development Branch of the US National Institute of Allergy and Infectious Diseases.

Ogobara Doumbo, MD, PhD
Director of the MRTC

____
Signature

____
Date

PRINCIPAL INVESTIGATOR'S STATEMENT:

I, the undersigned, have reviewed this protocol, including Appendices, and will conduct the clinical study as described and will adhere to the principles of the ICH/GCP as well as all applicable regulatory requirements. I have read and understood the contents of the Investigator's Brochure provided by the Malaria Vaccine Development Branch of the US National Institute of Allergy and Infectious Diseases.

Alassane Dicko, MD, MS

____
Signature

____
Date

Protocol Summary

Title: Randomized, Controlled Phase 1/2 Study of the Safety and Immunogenicity of AMA1-C1/Alhydrogel® Vaccine for *Plasmodium falciparum* Malaria, in Children in Donéguébougou and Bancoumana, Mali

Study Population: Healthy, malaria-exposed male and female volunteers aged 2-3 years, inclusive.

Rationale: Apical membrane antigen-1 (AMA1) is a surface protein expressed during the asexual blood stage of *P. falciparum*. It is produced as an 83-kDa polypeptide by mature schizonts in infected erythrocytes. In clinical studies in malaria-unexposed adults in the USA and in malaria-exposed adults in Mali, AMA1-C1/Alhydrogel® was safe and immunogenic. This study will evaluate its safety and immunogenicity in malaria-exposed children living in an area of seasonal malaria transmission.

Study Design:

- Randomized, active-controlled Phase 1 and Phase 2 clinical trial.
- Study centers: Donéguébougou (Phase 1) and Bancoumana (Phase 2), Mali.
- Number of volunteers: 336 in three cohorts
  - **<u>Cohort 1:</u>** Two dose-groups of 18 children each (20 µg or 80 µg AMA1-C1/Alhydrogel®), randomized 2:1 to receive either AMA1-C1/Alhydrogel® vaccine or the *Haemophilus influenzae* type B vaccine (Hiberix™) as active Comparator in a Phase 1 safety study in Donéguébougou.
  - **<u>Cohort 2:</u>** One group of 60, randomized 1:1 to receive either AMA1-C1/Alhydrogel® or Comparator (Hiberix™) in the staggered commencement of a Phase 2 study in Bancoumana.
  - **<u>Cohort 3:</u>** One group of 240, randomized 1:1 to receive either AMA1-C1/Alhydrogel® or Comparator (Hiberix™) in a Phase 2 study in Bancoumana.
- Study duration: Approximately 75 weeks; each volunteer will be followed for a maximum of 52 weeks.
- Immunization schedule: Vaccine Study Days 0 and 28.
- Route: IM in the thigh muscle.
- Dose of AMA1-C1: 20 and 80 µg for the first and second group of Cohort 1, respectively. Cohorts 2 and 3 will receive 20 µg or 80 µg depending on the safety results of Cohort 1.
- Dose of Alhydrogel®: 800 µg for each dose of AMA1-C1 vaccine.
- Cohort 1 will be followed for frequency of adverse events as well as episodes of infection with *Plasmodium falciparum* and associated disease throughout the 2006 transmission season for safety only.
- Cohorts 2 and 3 will be followed for frequency of adverse events as well as episodes of infection with *Plasmodium falciparum* and associated disease throughout the 2006 transmission season for safety and to measure the impact of vaccination on parasitological outcome.

Immunization Schedule:

Center and Week	Cohort 1		Cohort 2	Cohort 3
	Group 1	Group2		
Don0	12 A + 6 C			
Don2		12 B + 6 C		
Don4	12A + 6 C			
Don6		12 B+ 6 C		
Ban0			30 B+ 30 C	
Ban4			30 B+ 30C	
Ban8				120 B+ 120 C
Ban12				120 B+ 120 C

Don: Donéguébougou, Mali

Ban: Bancoumana, Mali

A: 20µg AMA1-C1/Alhydrogel®

B: 80µg AMA1-C1/Alhydrogel®

C: Comparator vaccine (Hiberix™)

Objectives:

Primary

1. To estimate the frequency of vaccine-related AEs, graded by severity, for each dose.
2. To determine the decrease in the number of parasitic episodes with a parasitemia >3000 per µL in subjects vaccinated with AMA1-C1 compared to subjects vaccinated with comparator vaccine.

Secondary

1. To describe the kinetics of the antibody response to AMA1-FVO and AMA1-3D7 following vaccination and during subsequent infection, as measured by enzyme-linked immunosorbent assay (ELISA).
2. To determine the AMA1 genotypes of parasites present following vaccination with AMA1-C1/Alhydrogel® or comparator vaccine.
3. To examine the vaccine effect on other parasitological and disease measures, as the basis for future Phase 2 malaria vaccine design.
4. To measure the inhibition of parasite growth by the *in vitro* growth inhibition assay (GIA) to parasite lines FVO and 3D7.
5. To determine the relationship between anti-AMA1 antibody concentrations, as judged by ELISA, and degree of *in vitro* growth inhibition of *P. falciparum*.

Product Description: The AMA1-C1 vaccine preparations to be studied contain an equal mixture of AMA1 from two different lines of *Plasmodium falciparum* (FVO and 3D7), both produced separately as recombinant proteins expressed by *Pichia pastoris*

(AMA1 FVO and AMA1 3D7). Purified AMA1 FVO and AMA1 3D7 were subsequently mixed and adsorbed onto the adjuvant aluminum hydroxide gel (Alhydrogel®).

The comparator vaccine is the licensed Hiberix™ *Haemophilus influenzae* type B vaccine (GlaxoSmithKline), a purified polyribosylribitol phosphate (PRP) capsular polysaccharide of *Haemophilus influenzae* type B covalently bound to tetanus toxoid.

1.0 INTRODUCTION

1.1 Background

1.1.1 Malaria as a Public Health Problem

As reported by the World Health Organization in 2002, the worldwide incidence of malaria is approximately 213 million clinical cases annually, with between 703 thousands to 1.6 million deaths per year attributed to malaria [1]. More than 50% of morbidity and mortality occur among children under 5 years. About 86% of the total mortality occurred in Sub-Saharan Africa. Of the four species of malaria that infect humans, *Plasmodium falciparum* is responsible for the majority of these deaths. Mounting drug resistance of the malaria parasite, as well as widespread resistance of mosquitoes to insecticides make these control strategies increasingly inadequate. A vaccine that would reduce both mortality and morbidity secondary to *P. falciparum* infection would be a valuable new resource in the fight against this disease.

1.1.2 Disease Burden in Mali

In Mali, malaria is the leading cause of mortality and morbidity in the general population [2]. However, there are large geographical differences in malaria infection rates and in disease prevalence and incidence, depending on a variety of factors such as the climate (e.g., the amount of rainfall and the duration of rainy season), the type of agriculture practiced (e.g., rice versus millet production), and access and utilization of health care and protective measures (e.g., insecticide-treated bed nets). Malaria transmission in Mali is highly seasonal, occurring primarily during the rainy season, which lasts between 3 to 6 months, depending on the geographic location within Mali.

The prevalence of *Plasmodium* infection (as determined by microscopy) often exceeds 70% in children aged 2 to 9 years during the rainy season. According to epidemiological data collected by the Département d'Epidémiologie des Affections Parasitaires (DEAP) of the University of Bamako, the incidence of clinical malaria varies between 1.5 to 2 episodes per child per year, with some children experiencing up to 5 episodes of clinical malaria per year. Furthermore, the National Malaria Control Program (NMCP) of Mali reports that malaria fever represents 34% of all outpatient consultations in the country. Severe malaria accounts for 15% of hospitalizations in children between the ages of 0 and 14 years in the capital city of Bamako, with case fatality rates of approximately 17% at the National Pediatric Hospital in Bamako, versus 25% countrywide [3-6]. The most common presentation of severe malaria is cerebral malaria (61 to 84% of cases), whereas severe anemia is the cause of 8 to 30% of cases [4, 5].

1.2 The AMA1 Protein

Several *P. falciparum* antigens have been identified as potential vaccine components [7]. Proteins expressed by *P. falciparum* are generally specific to one stage of the parasite's life cycle. The apical membrane antigen-1 (AMA1) is a surface protein expressed during the asexual blood stage of *P. falciparum*. AMA1 is produced as an 83 kDa polypeptide by mature schizonts in infected erythrocytes [8], and localizes in the microneme, an

apical secretory organelle of the merozoite containing ligands for binding red cell receptors [9]. The protein is processed to a 66 kDa protein that is subsequently exported to the merozoite surface at around the time of rupture of the schizont-infected erythrocyte [10]. Although its exact function remains undetermined, these observations suggest that it performs a role during merozoite invasion of erythrocytes. Further evidence supporting this comes from studies with monoclonal antibodies to primate and murine plasmodia AMA1 that exhibit in vitro inhibition of parasite invasion of erythrocytes [11, 12]. This invasion inhibition is not a result of parasite agglutination by antibody, as it can be demonstrated that even the Fab fragments of these monoclonal antibodies block such invasion [13].

P. falciparum AMA1 consists of a signal sequence, a large extracellular domain (ectodomain), a transmembrane domain, and a short cytoplasmic tail. In comparison to nonhuman primate and mouse malaria parasites, *P. falciparum* AMA1 has a 45 amino acid extension after the signal sequence that is missing from all other *Plasmodium* species except the *P. falciparum*-like simian parasite *P. reichenowi*. Comparisons between all of the known amino acid sequences of AMA1 homologues indicate greater than 50% sequence identity, with 16 cysteine residues conserved in all sequences [14, 15, 16]. All of the cysteines are found in the ectodomain of the molecule, which is stabilized by eight intramolecular disulfide bonds [17].

AMA1 lacks the sequence repeats observed in other malaria antigens such as the merozoite surface antigens MSP1 and MSP2. However, sequence polymorphism resulting from point mutations is observed among alleles of the single copy AMA1 gene in *P. falciparum* [15, 18]. Escalante *et al*. compared AMA1 sequences from a total of 44 *P. falciparum* isolates from Kenya, India, Thailand, and Venezuela and observed polymorphism at 118 out of 622 amino acids [19]. No insertion/deletion mutations were observed, although approximately 70% of the mutations in the gene encoding AMA1 result in non-synonymous substitutions, suggesting positive natural selection. Evidence from this study and previous ones indicate that mutations are predominantly clustered within three regions of the ectodomain that are defined by the eight disulfide bonds and affect both B and T-cell epitopes [15].

Studies in Donéguébougou, Mali, have demonstrated that natural antibodies exist in a large proportion of individuals (>90%) in this area of intense, seasonal transmission. In a cross-sectional study of 200 individuals aged 6 months to 45 years, conducted in 2002 and 2003, median anti-AMA1 antibody levels reached approximately 1000 units/mL at the peak of malaria transmission. By approximately 4-years of age, children had attained adult levels of antibody [20].

Not only have natural antibodies to AMA1 been demonstrated in people living in malaria-endemic areas, these antibodies have also been shown to inhibit the *in vitro* growth of *P. falciparum* [21]. In this study, human anti-AMA1 IgG was affinity purified from a pool of plasma obtained from Papua New Guinean blood donors who had previously been found to have high titers of antibodies to a variety of *P. falciparum* asexual blood-stage antigens. When tested in the *in vitro* merozoite invasion assay, the affinity-purified human anti-AMA1 antibodies inhibited various strains of *P. falciparum*

in a manner similar to that observed with rabbit IgG raised to refolded *P. falciparum* AMA1 3D7. This inhibition was both dose-dependent and strain-specific. From this evidence, it is reasonable to postulate that boosting the natural antibody response to AMA1 through vaccination may protect an individual from illness due to the asexual blood stage of *P. falciparum* infection.

1.2.1 AMA1-C1 Vaccine

The AMA1-C1 vaccine being tested in this study contains an equal mixture of the correctly folded ectodomain portion of recombinant AMA1 from two different lines of *P. falciparum*: the FVO and 3D7 lines. The proteins AMA1 FVO and AMA1 3D7 were expressed separately as secreted recombinant proteins in *Pichia pastoris*, purified, and then combined in equal amounts by mass. A combination vaccine was chosen because of concerns about parasite polymorphism and evidence for strain-specific protection as outlined above. These two strains were chosen because their sequences are sufficiently different, when comparing all sequenced *P. falciparum* lines [19]. The intent is to provide a broad range of protection against the different strains of the parasite occurring in the field. Although relatively different among all known AMA1 sequences, the two recombinant proteins in the vaccine remain more than 95% homologous, the cysteine residues are in identical positions, and both are produced in the same expression system (*P. pastoris*). Additionally, the fermentation and purification of the two proteins are performed by procedurally similar batch production records.

Further evidence that combining two forms of AMA1 will help overcome the polymorphism of the AMA1 allele and thus provide broader protection in the field comes from studies that the Malaria Vaccine Development Branch (MVDB) has completed in rabbits. When immunized with a combination of recombinant AMA1 FVO and AMA1 3D7, titers of antibody to each individual antigen were similar to the titers obtained in rabbits immunized with each antigen separately [22]. Furthermore, antibody from those animals immunized with the combination vaccine (AMA1-C1), when mixed with both 3D7 and FVO parasites *in vitro*, inhibited parasite growth equally. When sera from rabbits immunized with only a single form of AMA1 were tested against the heterologous parasite, considerably less inhibition was seen than against the homologous parasite.

1.3 AMA1-C1 Vaccine Description

1.3.1 AMA1

Both recombinant AMA1 FVO and AMA1 3D7 are highly purified 62 kDa proteins that correspond to the ectodomain of *P. falciparum* (FVO) AMA1 and *P. falciparum* (3D7) AMA1, respectively. Both forms of AMA1 consist of amino acids 25 through 545 of the published sequences of each line's AMA1 gene (GenBank accession number AJ277646 for FVO and accession number U65407 for 3D7). AMA1 FVO and AMA1 3D7 each consist of the ectodomain of the mature protein found in parasites with the addition of a 6-histidine C-terminal tag to allow purification of the protein. The proteins AMA1 FVO and AMA1 3D7 were expressed separately as secreted recombinant proteins in *P. pastoris* and purified by a combination of affinity, ionic, hydrophobic, and gel filtration chromatography.

1.3.2 *Alhydrogel®*

Aluminum hydroxide gel (HCI Biosector, Denmark) has been extensively used as an adjuvant in many licensed human vaccines. Aluminum-containing adjuvants are in routine human use and contained in many licensed human vaccines.

1.3.3 *Vaccine Production & Formulation*

The synthetic AMA1 gene sequences were subcloned into the *P. pastoris* expression plasmid, pPIC9K (Invitrogen Corporation, Carlsbad, California), which encodes a pre-pro-secretory α-factor sequence that is cleaved by the yeast enzyme KEX2 during protein maturation. AMA1 FVO and AMA1 3D7 were purified from the fermentation supernatant using a combination of affinity, ionic, hydrophobic, and gel filtration chromatography. The purification process was designed to separate full-length product from degraded material as well as non-product-related contaminants. AMA1 FVO and AMA1 3D7 bulk antigens (drug substances) were both manufactured at the WRAIR Bioproduction Facility (Silver Spring, Maryland) according to cGMP. Immediately prior to formulation, equal weights of AMA1 FVO and AMA1 3D7 were mixed and then bound to Alhydrogel®. AMA1-C1 refers to the mixture of AMA1 FVO and AMA1 3D7. The AMA1-C1/Alhydrogel® vaccine refers to AMA1-C1 formulated on Alhydrogel®.

1.4 Clinical Experience with AMA1-C1/Alhydrogel® Vaccine

To date, four adult Phase 1 trials have included AMA1-C1/Alhydrogel®. These trials are detailed in the Investigator's Brochure and are summarized in **Table 1** below.

Table 1 Summary of AMA1-C1/Alhydrogel® Phase 1 Trials

Trial	No. of Volunteers	Dose	Adjuvant	Schedule	Comments
Johns Hopkins University, 2003	10	5 µg	Alhydrogel®	0, 1 and 6 months	No vaccine related safety concerns were observed
	10	20 µg			
	10	80 µg			
Donéguébougou, MRTC, Bamako, 2004	12	5 µg	Alhydrogel®	0, 1 and 12 months	No vaccine related safety concerns were observed
	12	20 µg			
	12	80 µg			
University of Rochester, 2005	20	20 µg	Alhydrogel® + CPG 7909	0, 1 and 2 months	15 volunteers received 20 µg AMA1-C1/Alhydrogel® + CPG 7909 and 5 volunteers have received 80 µg AMA1-C1/Alhydrogel®
	20	80 µg	Alhydrogel® + CPG 7909		15 volunteers received 80 µg AMA1-C1/Alhydrogel® + CPG 7909 and 5 volunteers have received 80 µg AMA1-C1/Alhydrogel®
	0 of 35 planned	80 µg	Alhydrogel® + CPG 7909		15 volunteers will receive 80 µg AMA1-C1/Alhydrogel® + CPG 7909 and 20 volunteers will receive 80 µg AMA1-C1/Alhydrogel®
Clinical Center, NIH, 2005	3 of 18 planned	80 µg	Alhydrogel®	0, 1 and 2 months	3 volunteers received 80 µg AMA1-C1/ Alhydrogel® of 12 planned and 6 will receive Alhydrogel® alone

No safety concerns relating to the use of AMA1-C1/Alhydrogel® have been found in any of these trials. The first Phase 1 trial showed that the vaccine was immunogenic in naïve adults. The Mali trial also observed a significant increase in antibody, especially in the 80 µg groups.

1.5 Clinical Development Plan

The clinical development plan for AMA1-C1/Alhydrogel® in children is detailed in the Investigator's Brochure. Briefly the following trials are envisaged as lying on the critical path for children.

- USA 2003: Phase 1 study for safety and immunogenicity in naïve adults
- Mali 2004: Phase 1 study for safety in adults.
- Mali 2006: Phase 1/2 study for safety in 2-3 year old children (this protocol).
- Mali 2007: Extended Phase 2 studies for safety and impact on parasitemia in 2-3 year children in endemic areas.
- Phase 3 studies to measure impact of vaccine on severe morbidity in children due to falciparum malaria.

1.6 Comparator Vaccine

1.6.1 Rationale for Use of a Comparator Vaccine

Having a control group is essential to compare frequencies of adverse events in both the Phase 1 and Phase 2 parts of this study, as well as the number of episodes of parasitemia >3000 per µL that constitute the biological end point for the Phase 2 component. The control group is also important for the secondary outcome to describe the antibody kinetics (since children will make antibody as a response to both the vaccine and malaria infection) and to determine if vaccinated subjects are infected with parasites with a different AMA1 genotype than non-vaccinated subjects.

1.6.2 Rationale for Use of the **Haemophilus influenzae** *type B (Hib) Vaccine as a Comparator*

We have chosen Hiberix™ (Glaxo SmithKline) as the comparator vaccine for the following reasons: it is likely to confer some benefit to the volunteers receiving it, it has a proven safety record, and its dosing schedule permits incorporation into the study design.

Haemophilus influenzae type B vaccine was just introduced into the Expanded Program for Immunization (EPI) in Mali in 2005 and has been limited to the large cities. Most children living in rural Mali including Donéguébougou and Bancoumana have not received *Haemophilus influenzae* type B (Hib) vaccine. A recent study in Mali has found a substantial burden of Hib disease in children of 0-5 years of age with an annual incidence of $45.2/10^5$ [23].

The recommended dosing schedule for Hiberix™ for children of 2 to 12 months of age is to give three 0.5mL doses at an interval of at least four weeks between the doses. Although a single dose is considered enough for children 13 months of age and above, a booster dose provides additional benefit without an increase in systemic adverse

events.[24]. Study participants randomized to one of the AMA1-C1/Alhydrogel® arms of the study will be offered Hiberix™ free-of-charge after the conclusion of the study.

2.0 OBJECTIVES

2.1 Primary Objective

1. To determine the frequency of vaccine-related AEs, graded by severity, for each dose
2. To determine the decrease in the number of parasitic episodes with a parasitemia >3000 per µL in subjects vaccinated with AMA1-C1 compared to subjects vaccinated with Hiberix™ comparator vaccine.

2.2 Secondary Objectives

1. To describe the kinetics of the antibody response to AMA1-FVO and AMA1-3D7 following vaccination and during subsequent infection, as measured by enzyme-linked immunosorbent assay (ELISA).
2. To determine the AMA1 genotypes of parasites before and following vaccination with AMA1-C1/Alhydrogel® or comparator vaccine.
3. To examine the vaccine effect on other parasitological and disease measures, as the basis for future Phase 2 malaria vaccine design.
4. To measure the inhibition of parasite growth by the *in vitro* growth inhibition assay (GIA) to FVO and 3D7.
5. To determine the relationship between anti-AMA1 antibody concentrations, as judged by ELISA, and degree of *in vitro* growth inhibition of *P. falciparum*.

3.0 STUDY SITES

The study will be conducted in two rural villages, Donéguébougou and Bancoumana in Mali. Donéguébougou has a population of 1400 inhabitants and is located 30 km northwest of Bamako, the capital of Mali. Bancoumana is located at 60 km southwest of Bamako and has a population of about 10,000 people. Both sites are situated in the Sudanian area of Mali. The climate is hot, with daily temperatures ranging from 19 to 40°C. The annual rainfall varies between 600 and 1200 mm from June to October.

Intensive epidemiological longitudinal surveys have been undertaken in Donéguébougou since 1999 [25]. These data show that Donéguébougou is a suitable village for conducting Phase 1 and Phase 2 malaria vaccine trials and the data of infection frequencies in 1999 and 2000 provided the basis for the power calculations in this study. The Malaria Research and Training Center (MRTC) has established a clinical trial center in the village and one AMA1-C1 vaccine trial has taken place in Donéguébougou. Because of the excellent trial infrastructure and community involvement, Donéguébougou has been chosen as the site for the first Phase 1 study of AMA1-C1/Alhydrogel® in children.

Bancoumana has also been the subject of considerable epidemiological studies [26, 27] and has a similar epidemiology to Donéguébougou. The MRTC is in the process of

establishing a clinical trial center in Bancoumana and this will be functional prior to commencement of the Phase 2 studies. Bancoumana has a much larger population (about 10,000 inhabitants) than Donéguébougou and this larger population base will be required to enable sufficient subjects to be recruited for the Phase 2 component of this study.

4.0 STUDY DESIGN

4.1 Overall Design

This protocol describes the Phase 1 and Phase 2 clinical testing of AMA1-C1/Alhydrogel® in healthy malaria-exposed Malian children.

The first part of this study is a randomized, controlled Phase 1 clinical trial. Thirty-six healthy Malian children in Donéguébougou will be enrolled and randomized 2:1 to receive two doses 4 weeks apart of AMA1-C1/Alhydrogel® at 20 or 80 µg , or a comparator vaccine. As the primary purpose of the 20 µg group is to establish that the vaccine is safe enough to test in the 80 µg group, prior to dose escalation, safety data up to and including Day 7 post-vaccination from the 20 µg group Dose 1 will be reviewed by the Medical Monitor. The trial will not proceed to vaccination of the 80 µg group if, in the clinical judgment of the Medical Monitor, the 80 µg dose of AMA1-C1 would pose an unacceptable safety risk to the volunteers.

The decision to proceed to Phase 2 testing in malaria-exposed children will be taken after the interim safety analysis of this study has been reviewed by the Investigators, the Medical Monitor, the Data and Safety Monitoring Board (DSMB), the sponsor and the IRBs of the University of Bamako and the National Institute of Allergy and Infectious Diseases (see **Section 9.2.1**). The above reviews will be conducted by the DSMB, NIAID and Mali IRBs according to the schedule and table described in **Section 8.6**. The interim analysis will include the safety data of all volunteers up to 7 days after the second immunization with the 80µg dose (Day 35 of trial). The interim analysis will not include immunogenicity data. The choice of the dose for the Phase 2 will be based on the safety results. The study will proceed to the Phase 2 part with the 80 µg dose unless an important safety issue was identified. The investigators will remain blinded during and after the interim safety analyses (see **Section 4.5**). Documentation from reviews by the Medical Monitor, the DSMB, the sponsor, and the IRB will be provided to the NIAID IRB.

The second part of this study is a Phase 2 trial of AMA1-C1/Alhydrogel® in Malian children in Bancoumana, Mali. The study will be a double-blinded, randomized, controlled, Phase 2 clinical trial in healthy, malaria-exposed children volunteers designed to evaluate the safety, reactogenicity, and immunogenicity of the AMA1-C1 malaria vaccine formulated on Alhydrogel®, as compared to the comparator vaccine Hiberix™. In this part of the study, two cohorts with a total 300 volunteers will be enrolled in a progressive fashion. The first cohort of subjects at Bancoumana (Cohort 2 of the study) allows for a more extensive safety evaluation of the vaccine prior to moving into larger group sizes, and will enroll 60 volunteers, randomized 1:1 to receive two immunizations 4 weeks apart of AMA1-C1/Alhydrogel® at 80 µg dose or the comparator vaccine. After

Day 35 for Cohort 2, the randomization code will be revealed to the DSMB and an interim report detailing the safety (see **Sections 8.6** and **9.2.1**) will be prepared. This safety report will form the basis for decisions to proceed with Cohort 3 of 240 volunteers which will be double-blinded and randomized 1:1 to receive two immunizations 4 weeks apart of AMA1-C1/Alhydrogel® at 80 µg dose or the comparator vaccine.

The second vaccination of Cohort 3 must be completed before the end of August, *i.e.*, soon after the commencement of rains which mark the start of the transmission season in Donéguébougou and Bancoumana.

A similar vaccination and examination schedule will be used for all subjects in all three cohorts. After obtaining the consent and cooperation of the village leaders and local officials, subjects in the target age group (2-3 years) will be invited to participate in the study. After obtaining written informed consent from a parent or legal guardian, subjects will undergo eligibility screening, including medical history and physical examination, hematology testing, liver and renal function testing, Hepatitis B and C screening, and urinalysis. All clinically significant abnormalities will be reviewed with each volunteer's parent or legal guardian and referral for follow-up care will be provided. After screening, those volunteers determined to be eligible, based on the inclusion and exclusion criteria described in **Section 5.0** in this protocol, will be invited to participate in the study.

All subjects will be vaccinated at time 0 and at 4 weeks. As with other aluminum hydroxide-adsorbed vaccines, hypersensitivity reactions would be expected to occur within the first 24 hours after receipt of either of the two vaccines, and other severe local or systemic reactions within 72 hours of vaccination. Subjects will therefore be observed for immediate reactions following each vaccination for 30 minutes with emergency equipment readily available, and will return to the study clinic on Days 1, 2, 3, 7, and 14 following each vaccination for clinical assessment. See **Table 2** for a tabular description of the vaccination schedule for the two dose cohorts, as well as **Section 7.6** and **Appendices B and C** for a detailed description of the scheduled clinical and laboratory evaluations.

All subjects will be examined at weekly intervals through the transmission season commencing 2 weeks following the commencement of the second vaccination of the 3rd cohort. At the first post-vaccination surveillance visit and every subsequent 4 weeks, subjects will have a blood sample taken for hemoglobin measurements, for thick and thin blood slides, to enable genotyping of AMA1 if infected and for AMA1 ELISA. At every weekly visit, subject and parents will be questioned about the subject's state of health. If there is evidence of current or recent malaria infection (temperature $>37.5^{o}C$ at the time of visit or history of fever in the preceding week) then a blood sample will be taken. The slide will be read immediately and if parasitemic the subject will be treated according to the guidelines of the National Malaria Control Program in Mali. Investigators will be blinded to the vaccine allocation for participants in Cohort 1 until the Day 42 visit. After that point, the code will be revealed to the investigators to allow a clear assessment of the safety in each group and this part of the study will be single blinded (*i.e.*, study participants will remain blinded to what vaccine they have received).

The investigators will be blinded to the vaccine allocation status for participants in Cohorts 2 and 3 until the conclusion of the transmission season. The study duration will be a maximum of 75 weeks and each volunteer will be followed for 52 weeks. Both Cohorts 2 and 3 (total of 150 vaccinated receiving AMA1-C1/Alhydrogel® and 150 receiving the comparator vaccine) will be used to evaluate the impact of the vaccine on parasitemia.

4.2 Sample Size and Estimated Duration of Study

A total of 336 volunteers will be enrolled in three cohorts (36, 60 and 240, respectively). Detailed justification of the sample size is given in **Section 10.2**. The trial is expected to last for a maximum of 75 weeks and each volunteer will be followed for a maximum of 52 weeks from the time of the first injection.

4.3 Timing of Trial

Malaria transmission in Mali is seasonal and coincides with the wet season. Most of the transmission commences in July and ends in December. Therefore, timing of vaccinations in this protocol is critical. Specifically the second vaccination for Cohort 3 should not be earlier than the end of June or later than the end of August. Ideally, the second vaccination should occur by the end of July and this would allow parasitological follow up over the complete transmission season. However, in order to allow as much latitude as possible, the study has been powered on the assumption that the minimum parasitological follow up will occur over three months of intense transmission: September, October and November. See **Section 10.2**. In order for the trial to be adequately powered, a total of 900 follow-up person-months among the 300 volunteers is required.

4.4 Group Allocation

4.4.1 Phase 1 Study

This Phase 1 study will be conducted in Donéguébougou and will have two dose groups that will be enrolled consecutively. These two dose groups will constitute the first cohort (Cohort 1). Within each group of 18 volunteers of that cohort, 12 will be randomly assigned to receive AMA1-C1/Alhydrogel® vaccine and 6 to receive the comparator vaccine (see **Section 7.3**). Within the Group 1 of the first dose cohort ("Cohort 1, Group 1"), those randomized to receive the AMA1-C1/Alhydrogel® vaccine will receive that which contains 20 µg, whereas within the second group of that cohort ("Cohort 1, Group 2"), those randomized to receive the AMA1-C1/Alhydrogel® vaccine will receive that which contains 80 µg of AMA1-C1.

Group 1 of Cohort 1 will be assembled first. Once screening has started and the first 20 participants have been deemed eligible, they will be assigned to Group 1 and a Day 0 visit will be scheduled. On the day of first vaccination, the first 18 who arrive at the study clinic will be randomly assigned in a 2:1 fashion to receive either the AMA1-C1/Alhydrogel® vaccine (20 µg) or comparator vaccine; the remaining 2 will be kept as alternates if some of the first 18 cannot be vaccinated on the day of first vaccination (*i.e.*, withdrawal of consent, acute disease, *etc.*). If these alternates are not vaccinated, they will be invited to participate as members of the second group of Cohort 1.

Following assembly of Group 1 of Cohort 1, the next 20 eligible volunteers screened will be assigned to Group 2 and their Day 0 visits scheduled using the same procedure as described for Group 1. The alternates from this cohort will be considered screened but not enrolled. (see **Section 7.4**)

4.4.2 Phase 2 Study

This Phase 2 study will be conducted in Bancoumana and will include 2 cohorts (Cohort 2 and Cohort 3) pending approval of the DSMB after review of the interim safety report (including up to Day 35 of the second group of Cohort 1) of the Phase 1 study. Cohort 2 will be assembled and vaccinated first. After the Day 35 visit of Cohort 2, a safety report will be prepared and reviewed by the DSMB. Upon approval of the DSMB, new subjects will be screened for enrollment into the third cohort (Cohort 3).

Screening and Enrollment into Cohort 2

Children in the target age groups in Bancoumana will be invited to participate in the study. If the invitation is accepted, informed consent will be obtained from parents and then the children will be screened. Once 70 eligible children are identified, the immunization days for that cohort will then be scheduled. The cohort will be divided into two groups. A first group of 30 subjects will be vaccinated followed by a second group of 30 subjects 4 days later. On the day of the first immunization of the first group, 35 subjects will be invited and the first 30 will be randomized in 1:1 ratio to receive the AMA1-C1/Alhydrogel® vaccine or the comparator vaccine; the remaining 5 will be kept as alternates if some of the first 30 cannot be vaccinated on that day (i.e., withdrawal of consent, acute disease, etc.). The alternates that are not vaccinated will be invited to participate as members of second group. On the day of first vaccination of the second group, the 5 remaining subjects from the first group will be invited and the first 30 subjects will be randomized in 1:1 ratio to receive the AMA1-C1/Alhydrogel® vaccine or the comparator vaccine.

Screening and Enrollment into Cohort 3

Cohort 3 will be constituted only after DSMB approval following review of the safety data of Cohort 2 including up to Day 35 visit. Once the DSMB approval is obtained, subjects in the target age groups in Bancoumana not yet enrolled in the study will be invited to participate. If the invitation is accepted, informed consent will be obtained from parents and then children will be screened for inclusion and exclusion criteria. Once 250 children eligible are identified, the immunization days for that cohort will then be scheduled. The cohort will be divided into groups of 20 to 40 subjects to be vaccinated the same day. In each group, subjects will be randomized in 1:1 ratio to receive the AMA1-C1/Alhydrogel® vaccine or the comparator vaccine. Alternates from Cohort 2 may enroll into Cohort 3 but will not be given preference and will have to be re-screened, as there will be an 8 week time lapse between the cohorts.

4.5 Blinding

Subjects will be blinded as to their allocation to either AMA1-C1/Alhydrogel® or comparator vaccine until the end of the study. Due to the staggered, dose-escalation design of the trial, it will not be possible to blind participants in Cohort 1 to the dose of AMA1-C1 that they may have received. For cohorts 2 and 3, with the exception of the statistician and/or his delegate and pharmacists, all other investigators and their staff will be blinded to the results until the database is cleaned and locked following the end of the parasitological follow up (**Section 7.6.2**). The Medical Monitor will be supplied with the sealed copy of the randomization code that can be used if necessary for investigation of adverse events. The DSMB will be supplied with three unblinded interim reports generated by the study statistician detailing frequency of adverse events categorized by group. These reports provide the data for recommendations by the DSMB to the IRBs, sponsor, and investigators for proceeding with vaccination of Cohort 2, Cohort 3, and with the continuation of the parasitological follow up. These reports may be circulated to the IRBs but unless there are serious safety concerns, these unblinded reports must not be circulated to the trial investigators. The DSMB summary recommendations will be distributed to the investigators and the IRBs. The investigators will be blinded to participants' vaccine allocation status until in January 2007 following the conclusion of the transmission season. Participants will be blinded as to their allocation to either AMA1-C1/Alhydrogel® or the comparator vaccine until the end of the study.

5.0 SELECTION AND ENROLLMENT OF VOLUNTEERS

5.1 Inclusion Criteria

1. Males or females aged 2 to less than 4 years old. Children must be born no earlier than September 1, 2002 and must have had their second birthday prior to first vaccination.
2. Known residents of the village of Donéguébougou, Mali (Cohort 1) or Bancoumana (Cohorts 2 and 3).
3. Good general health as determined by means of the screening procedures.
4. Available for the duration of the trial (52 weeks).
5. Willingness to participate in the study as evidenced by parents/legal guardians signing or fingerprinting the informed consent document.

5.2 Exclusion Criteria

1. Evidence of clinically significant neurologic, cardiac, pulmonary, hepatic, rheumatologic, autoimmune, chronic infectious or renal disease by history, physical examination, and/or laboratory studies including urinalysis.
2. Behavioral, cognitive, or psychiatric disease that in the opinion of the investigator affects the ability of the volunteer or the parent/legal guardian to understand and cooperate with the study protocol.
3. Laboratory evidence of liver disease (alanine aminotransferase [ALT] greater than 1.25 times the upper limit of normal of the testing laboratory).

4. Laboratory evidence of renal disease (serum creatinine greater than the upper limit of normal of the testing laboratory, or more than trace protein or blood on urine dipstick testing).
5. Laboratory evidence of hematologic disease (absolute leukocyte count <3000/mm^3 or >14,500/mm^3, absolute lymphocyte count <1000/mm^3, platelet count <120,000/mm^3, or hemoglobin <8.5 g/dL).
6. Other condition that in the opinion of the investigator would jeopardize the safety or rights of a volunteer participating in the trial or would render the subject unable to comply with the protocol.
7. Participation in another investigational vaccine or drug trial within 30 days of starting this study, or while this study is ongoing.
8. History of a severe allergic reaction or anaphylaxis.
9. Severe asthma (emergency room visit or hospitalization within the last 6 months).
10. Positive ELISA for HCV.
11. Positive HBsAg by ELISA.
12. Known immunodeficiency syndrome.
13. Use of corticosteroids (excluding topical or nasal) or immunosuppressive drugs within 30 days of starting this study.
14. Receipt of a live vaccine within past 4 weeks (e.g. measles/mumps/rubella (MMR)) or a non-live vaccine (e.g. diphtheria/pertussis/tetanus (DPT)) within past 2 weeks prior to entry into the study.
15. History of a surgical splenectomy.
16. Receipt of a blood transfusion within the past 6 months.
17. Previous receipt of an investigational malaria vaccine.
18. History of a known allergy to nickel.
19. History of known allergy to yeast.
20. Known hypersensitivity to any component of the Hib vaccine (tetanus toxoid, lactose)
21. Previous administration of Hib vaccines.
22. Known thrombocytopenia or bleeding disorders.

5.2.1 Rationale for Use of Clinical Assessments of Immunosuppression

We do not plan to test for HIV at the time of screening because the HIV seroprevalence is 1.7% in Mali, one of the lowest rates in sub-Saharan Africa. Although no serosurveys have been done in Donéguébougou and in Bancoumana, these sites are in a rural area and almost certainly have a lower prevalence rate than the average for the entire country. Therefore, the training of staff and establishment of programs that would be necessary for voluntary counseling and testing for HIV would likely yield few, if any, cases of HIV in the study. In case clinical immunosuppression is diagnosed during the screening or after enrollment, the subject will be referred to the health care center in charge of such therapies, and will be diagnosed and treated according to the National Guidelines using HAART. Access to treatment will be facilitated during the entire period of the study.

5.3 Treatments That Could Potentially Interfere with Vaccine-Induced Immunity

The following criteria will be checked at each visit. If any become applicable during the study, the participant will be excluded from receiving further doses of the study vaccine and will not be included in the parasitological and immunogenicity evaluations after the time of exclusion. The participant will, however, be encouraged to remain in the safety evaluation including monitoring of malaria infections for safety.

1. Use of any investigational drug or investigational vaccine other than the study vaccine during the study period.
2. Administration of chronic (defined as more than 14 days) immunosuppressants, corticosteroids, or other immune-modifying drugs 6 months prior to vaccination or while the study is ongoing. (Topical and nasal steroids are allowed.)
3. Administration of a licensed vaccine during the period starting from Day -14 to Day 42 (14 days before and after each vaccination).
4. Administration of immunoglobulins and/or any blood products up to 30 days after the last dose of vaccine.
5. Splenectomy during the course of the study.

5.4 Contraindications to Vaccination

The following criteria will be checked prior to each immunization and are contraindications to further immunization. However, the participant will be encouraged to remain in the safety evaluation for doses already received.

1. Hypersensitivity reaction following administration of the study vaccine.
2. Occurrence of severe disease, which in the view of the investigators or Medical Monitor could jeopardize the safety of the study participant or may complicate interpretation of the safety or immunogenicity data.

5.5 Indications for Deferral of Vaccination

The following adverse events (AEs) constitute grounds for deferral of vaccine administration at that point in time; if any one of these AEs occurs at the time scheduled for vaccination, the participant may be vaccinated at a later date, within the allowable time interval specified in **Section 7.6** of this protocol, or withdrawn at the discretion of the investigator. The participant must be followed until resolution of the event as with any AE. If the participant is withdrawn from the study, he/she will be encouraged to remain in the safety evaluation for the duration of the study.

1. Axillary temperature ≥ 37.5°C or other evidence of clinical malaria at the time of vaccination will warrant deferral of immunization until fever and symptoms resolve.
2. Any other acute condition that in the opinion of the investigator poses a threat to the individual if immunized or that may complicate interpretation of the safety of the vaccine following immunization.

Such individual(s) will be followed in the clinic until the symptoms resolve or the window for immunization expires. No further vaccination will be performed if the

participant does not recover (axillary temperature < 37.5°C and/or lack of symptoms) within the originally scheduled vaccination time interval. The participant, however, will be followed for safety and immunogenicity evaluations. If the individual meets any of the above criteria for deferral on the day of first immunization, as an alternative to deferral of vaccination, the investigator may instead elect to exclude the participant from further participation in the study. Eligible alternates will then be vaccinated instead.

5.6 Subject Withdrawal Criteria

A volunteer will not complete the series of immunizations if any of the following reasons apply. However, any volunteer who has received at least one dose of vaccine will be encouraged to remain in the safety evaluation for the duration of the study.

1. *Research terminated by sponsor or investigator* – applies to the situation where the entire study is terminated by the sponsor, or investigator for any reason.
2. *Withdrawal of consent* – applies to a subject whose parents withdraw consent to participate in the study for any reason.
3. *Noncompliant with protocol* – applies to a volunteer who does not comply with protocol-specific visits or evaluations on a consistent basis, such that adequate follow-up is not possible and the volunteer's safety would be compromised by continuing in the trial. Additionally, this applies to a volunteer who is lost to follow-up and cannot be located.
4. *Developed an adverse event* - applies to a participant who is withdrawn from the study due to an adverse event, serious or otherwise.
5. *Lost to follow-up* - applies to a participant who consistently does not return for protocol study visits, is not reachable by any means of communication and/or is not able to be located or is absent from the village during parasitological follow up for a period longer than one week.
6. *Other* – is a category used when previous categories do not apply, and requires an explanation.

6.0 VACCINE PREPARATION

6.1 Supply of AMA1-C1/Alhydrogel®

The AMA1-C1/Alhydrogel® research products for this protocol will be supplied to the study-site pharmacist by the Pharmaceutical Development Section, Pharmacy Department, Clinical Center, National Institutes of Health, where the AMA1-C1/Alhydrogel® vaccine was formulated and vialed. Both the AMA1-C1/Alhydrogel® and Hiberix™ vaccines will be transported to Mali at 0.5°C to 9°C; temperature recording devices will accompany the vaccines at all times to ensure storage temperature limits have not been violated. Vaccine will be stored at the Faculté de Medicine, Pharmacie, et Odonto-Stomatologie (FMPOS) in Bamako in a refrigerator at 0.5°C to 9°C and will not be frozen; refrigerator temperature will be monitored continuously. One to two days prior to vaccination, adequate supplies of vaccine will be transported to the study site in temperature-monitored coolers; at the study site, they will be stored in a temperature-monitored refrigerator (powered by a generator with double backup). One day after all participants in a given cohort have been vaccinated, unused vials of vaccine will be

transported back to the FMPOS for storage until the next vaccination day. Single-dose vials will be stored in the upright position.

AMA1-C1/Alhydrogel® malaria vaccine is supplied as a slightly turbid suspension in single-dose vials. Each 2.0 mL vial contains a single dose, of which 0.5 mL is the intended volume to be injected. 0.5 mL of vaccine contains the equivalent of 424 µg of aluminum as Alhydrogel® (800 µg of aluminum hydroxide gel per dose) onto which either 20 µg or 80 µg of recombinant AMA1-C1 has been bound. The product conforms to established requirements for sterility, safety, and identity.

6.2 Supply of Comparator Vaccine

Hiberix™ is a noninfectious vaccine containing purified polyribosylribitol phosphate capsular polysaccharide (PRP) of Haemophilus influenzae type b covalently bound to tetanus toxoid. Hiberix™ is supplied as a white lyophilized pellet for reconstitution with sterile saline solution 0.9%. Each 0.5 mL dose contains 10 microgram of purified capsular polysaccharide of Hib covalently bound to approximately 30 microgram of tetanus toxoid. The Hiberix™ Hib polysaccharide is extracted from a culture of Haemophilus influenzae type b strain 20,752. After activation with cyanogen bromide and derivitization with an adipic hydrazide spacer, the Hib polysaccharide is coupled to tetanus toxoid via carbodiimide condensation. After purification, the Hib conjugate is lyophilized in the presence of a lactose stabilizer. Hiberix™ meets the World Health Organization requirements for the manufacture of biological substances and Hib conjugate vaccines.

6.3 Vaccine Storage

Both AMA1-C1/Alhydrogel® and Hiberix™ should be maintained at 0.5°C to 9°C until just prior to administration. Vaccine should NOT be frozen at any time.

6.4 Vaccine Accountability

Study-site pharmacists are responsible for maintaining an accurate inventory and accountability record of vaccine supplies for this study. Partially used vials may not be administered to other volunteers.

6.5 Disposition of Used/Unused Supplies

After administration of a vaccine dose, vials will be stored in the study pharmacy at the study site, and vials will be accounted for and stored until monitoring by the study sponsor. The vials may then be disposed of according to site protocol. At the conclusion of the study, <u>all</u> unused AMA1-C1/Alhydrogel® vaccine supplies will be destroyed on site, or returned to the Pharmaceutical Development Section, Pharmacy Department, Clinical Center, National Institutes of Health, who will return them to the Malaria Vaccine Development Branch (MVDB) or destroy them, as requested by the Sponsor. All other vials will be retained until the close out of the study and then can be destroyed on site.

7.0 STUDY PROCEDURES

The following sections provide a detailed listing of the procedures and studies to be performed for this protocol at designated time points. The total volume of venous blood (approximately 50mL) to be drawn over the 12-month duration of the trial should not compromise the health of trial participants.

7.1 Consent Procedures

The previous studies conducted by the MRTC in Donéguébougou and Bancoumana (as described in **Section 3.0**) have permitted extensive contact with the village population that has led to the development of mutual trust and the establishment of an ongoing informed consent process attempting to address issues related to interventional studies in resource-limited settings. Many discussions with village leaders, heads of families, school teachers, and villagers through group meetings and more limited group interviews have reviewed the need to obtain a written informed consent from study participants. The community has now become familiar with the informed consent process, including written, signed consent forms.

The community informed consent process goes through the following steps:

i. Explanation and clarification to village leaders, including the village chief and elders.
ii. Allow time for village leaders to communicate with community members and relay any additional questions or concerns.
iii. Take time to explain protocols to heads of families.

Prior to administering individual informed consent, the study team conducts careful word-for-word review of the study consent form that will be translated orally into local languages and dialects in the event that a potential study participant does not read or speak French (which will likely be a majority of the potential participants). Verification that the oral translations are accurate and that the potential participants understand the contents of the informed consent form will be done by the independent witness as described in **Section 7.2**.

7.2 Individual Recruitment and Informed Consent

Subjects in the target age group (2-3 years) will be invited to come to the study clinic for screening with their parents or guardians. During this initial screening visit, the parent or legal guardian of the potential participant will read the consent form or have it explained to him/her in cases of illiteracy. They will be encouraged to ask questions, and then take a multiple-choice questionnaire to evaluate consent comprehension (**Appendix A**); this will be administered orally to the parent or guardian of potential volunteers in case they cannot read. The parent or legal guardian must answer all questions correctly before the child becomes eligible for enrollment. Study staff will use incorrect answers from the questionnaire to identify those areas of the informed consent form that need further review with the parent or legal guardian. This will help ensure he/she has sufficient understanding before signing the consent form. He/she may either sign the consent form immediately or later after further consideration. A parent or legal guardian unable to read will place an imprint of his/her finger in the place of a signature; in addition, an

independent witness will sign the consent form to attest that the volunteer fully comprehended the contents.

The following procedures will be performed upon initial screening (note that all procedures might not be performed on the same day):

1. Explain the study and informed consent to the parent or legal guardian of the potential participant.
2. Ensure that the parent or legal guardian has passed the informed consent comprehension exam, that the consent document has been signed, and that a copy has been given to him/her.
3. Elicit a complete medical history
4. Administer a complete physical examination.
5. Obtain approximately 5 mL blood for hematology, biochemistry, and serologic tests for viral hepatitis.
6. Obtain urine for urine dipstick testing.

Any clinically relevant finding that is discovered upon screening will be treated appropriately according to the standard of care in Mali as follows. Initial management will be performed at the study clinic free of charge. Should referral for more extensive investigation or treatment be required, the study will arrange and pay for transportation to one of the National Hospitals and initial consultation. Initial care – according to the standard of care in Mali – will be covered by the study; however, in the event that a chronic illness is discovered during the course of screening, the study team will facilitate access to medical care, but long-term treatment and care will not be reimbursed by the study.

7.3 Randomization Process

The eligible volunteers assigned to the cohorts will be asked to come to the study clinic on their scheduled day of enrollment into the study. After undergoing a clinical interview with the child's parent or legal guardian and physical exam to ensure that they remain eligible for participation in the study, they will have blood collected for the studies outlined in **Section 7.6** and volunteers will be vaccinated as described in **Section 7.5**.

Study participants will be assigned a unique study number and will be given a photo identification card to aid in their identification; a copy of the photo identification card will be placed in the participant's study file.

Randomization to receive either AMA1-C1/Alhydrogel® or comparator vaccine will be done through use of a list of randomization codes. The randomization list will be prepared in advance of the start of the study and will contain sequential codes linking a study number to a vaccine assignment (AMA1-C1 or comparator vaccine). The study numbers will be assigned in the order in which the participants are enrolled in the study. For example, in Group 1 of Cohort 1, among the first 18 study numbers, 12 will be assigned to AMA1-C1 and 6 will be assigned to the comparator vaccine. Assignment of the study numbers will be done on the day of first vaccination, in the order that the study participants present for immunization.

7.4 Enrollment

Volunteers will not be considered enrolled in the study until they have received their first dose of vaccine.

7.5 Immunization Procedure

Volunteers will receive two immunizations, on Days 0 and 28. Both AMA1-C1/Alhydrogel® vaccine and Hiberix™ vaccine will be kept refrigerated at 0.5°C to 9°C until just before use, whereupon they will be warmed to room temperature. 0.5 mL (for the AMA1-C1/Alhydrogel® vaccine) or 0.5 mL (for the comparator vaccine) will be delivered by IM injection in the thigh muscle with a 22-gauge needle of appropriate length after preparation of the site with alcohol. Successive vaccinations will be given in alternating legs.

Participants who received AMA1-C1/Alhydrogel® vaccine will be offered the comparator vaccine at the end of the trial.

7.6 Clinical Monitoring and Evaluation

See **Appendices B and C** for a tabular representation of study procedures.
An identical procedure will be used for all subjects in all three cohorts.

7.6.1 Vaccination and Vaccine Follow Up

Vaccine Study Day 0 (Day of First Vaccination)

1. Verify that Informed Consent was obtained.
2. Verify that all applicable eligibility criteria have been met.
3. Perform abbreviated history and physical exam, focusing on any acute complaints. During the physical examination study staff will discuss signs and symptoms of potential AEs.
4. Obtain blood for hematology, biochemistry, GIA, anti-AMA1 antibody ELISA, malaria thin and thick smears and filter paper collection.
5. Record vital signs (blood pressure, temperature, heart rate, and respiratory rate).
6. Administer the vaccine.
7. Observe for 30 minutes after vaccination to evaluate for immediate adverse reactions.

Vaccine Study Day 1

1. Perform basic history and physical exam (including injection site), emphasizing examination of any acute complaints.
2. Record vital signs.

Vaccine Study Day 2

1. Perform basic history and physical exam (including injection site), emphasizing examination of any acute complaints.
2. Record vital signs.

Vaccine Study Day 3

1. Perform basic history and physical exam (including injection site), emphasizing examination of any acute complaints.
2. Record vital signs.
3. Obtain blood for hematology and biochemistry tests.

Vaccine Study Day 7 +/- 1

1. Perform basic history and physical exam (including injection site), emphasizing examination of any acute complaints.
2. Record vital signs.
3. Obtain blood for hematology.

Vaccine Study Day 14 +/- 2

1. Perform basic history and physical exam (including injection site), emphasizing examination of any acute complaints.
2. Record vital signs.
3. Obtain blood for hematology, biochemistry, malaria thin and thick smears, filter paper collection and anti-AMA1 antibody ELISA.

Vaccine Study Day 28 (from 28 to 35 days after the first vaccination)

1. Perform basic history and physical exam (including injection site), emphasizing examination of any acute complaints.
2. Obtain blood for hematology, biochemistry, malaria thin and thick smears, filter paper collection and anti-AMA1 antibody ELISA.
3. Record vital signs (blood pressure, temperature, heart rate, and respiratory rate). During the physical examination study staff will discuss signs and symptoms of potential AEs.
4. Administer the vaccine.
5. Observe for 30 minutes after vaccination to evaluate for immediate adverse reactions.

Vaccine Study Day 29 (1 day after Second Vaccination)

1. Perform basic history and physical exam (including injection site), emphasizing examination of any acute complaints.
2. Record vital signs.

Vaccine Study Day 30 (2 days after Second Vaccination)

1. Perform basic history and physical exam (including injection site), emphasizing examination of any acute complaints.
2. Record vital signs.

Vaccine Study Day 31 (3 days after Second Vaccination)

1. Perform basic history and physical exam (including injection site), emphasizing examination of any acute complaints.
2. Record vital signs.
3. Obtain blood for hematology and biochemistry tests.

Vaccine Study Week 5 (7 days +/- 1 day after Second Vaccination)

1. Perform basic history and physical exam (including injection site), emphasizing examination of any acute complaints.
2. Obtain blood for hematology.
3. Record vital signs.

Vaccine Study Week 6 (14 days +/- 2 days after Second Vaccination)
1. Perform basic history and physical exam (including injection site), emphasizing examination of any acute complaints.
2. Record vital signs.
3. Obtain blood for hematology, biochemistry, GIA, filter paper collection and anti-AMA1 antibody ELISA.

Vaccine Study Week 14 (10 weeks +/- 10 days after Second Vaccination)
1. Perform basic history and physical exam, emphasizing examination of any acute complaints.
2. Obtain blood for hematology, filter paper collection and anti-AMA1 antibody ELISA.

Vaccine Study Week 22 (18 weeks +/- 14 days after Second Vaccination)
1. Perform basic history and physical exam, emphasizing examination of any complaints.
2. Record vital signs.
3. Obtain blood for hematology, malaria thin and thick smears, filter paper collection, anti-AMA1 antibody ELISA, and GIA.

Vaccine Study Week 30 (26 weeks +/- 14 days after Second Vaccination)
1. Perform basic history and physical exam, emphasizing examination of any complaints.
2. Obtain blood for hematology, malaria thin and thick films, filter paper collection and anti-AMA1 antibody ELISA.

Vaccine Study Week 42 (38 weeks +/- 21 days after Second Vaccination)

1. Perform basic history and physical exam, emphasizing examination of any complaints.
2. Record vital signs.
3. Obtain blood for hematology, malaria thin and thick films, filter paper collection and anti-AMA1 antibody ELISA.

Vaccine Study Week 52 (48 weeks +/- 30 days after Second Vaccination)
1. Perform basic history and physical exam (including injection site), emphasizing examination of any acute complaints.
2. Record vital signs.
3. Obtain blood for hematology, malaria thin and thick films, filter paper collection, anti-AMA1 antibody ELISA, and GIA.

7.6.2 Parasitological Follow Up

Day 0 of the parasitological follow up for each subject will be determined from Day 42 of the first subject vaccinated in Cohort 3 as follows.

> Cohort 1: The last date prior to Cohort 3 Vaccine Study Day 42 from the following list: Cohort 1 Vaccine Study Week 18, 22, 26, 30 and 34. (Note: Cohort 1, Group 2 will be two weeks later than Cohort 1, Group 1).
>
> Cohort 2: Vaccine Study Week 14.
>
> Cohort 3: Vaccine Study Day 42 (Week 6).

The last day of the parasitology follow up for each subject will be determined as follows:

> Cohorts 1 and 2 will have the same number of planned parasitology follow up visits as Cohort 3.
>
> Cohort 3 last visit is the first time point in the following list after the 30th November 2006: Vaccine Study Week 12, 16, 20 and 24.

<u>Parasitology Study Day 0</u>
1. Record weight and height.
2. Perform basic history and physical exam (including temperature), emphasizing history of recent febrile episodes.
3. Make thick and thin malaria smears.*
4. Measure hemoglobin.*
5. Collect blood on filter paper for parasite typing.*
6. Collect 50 to 150 μL of blood for antibody.*
7. If axillary temperature ≥37.5^{o}C or history of recent fever, or Hemoglobin < 8.5 g/dL, read blood slides immediately and treat for malaria if parasite positive.

* by fingerprick if not previously collected as part of the Vaccine follow up

<u>Parasitology Study Week 1, 2, 3, 5, 6, 7, 9, 10, 11, 13, 14, 15, 17, 18, 19, 21, 22 and 23**</u>
1. Perform basic history and physical exam (including temperature), emphasizing history of recent febrile episodes.
2. If axillary temperature ≥37.5^{o}C or history of recent fever
    a. Collect blood sample by fingerprick for hemoglobin, malaria thin and thick films, filter paper collection and anti-AMA1 antibody ELISA.
    b. Immediately stain and read blood films.
    c. If blood film is parasite positive, begin malaria treatment.

** Study may terminate prior to Week 23 depending on starting date.

<u>Parasitology Study Week 4, 8, 12, 16, 20 and 24**</u>
1. Perform basic history and physical exam (including temperature), emphasizing history of recent febrile episodes.
2. Make thick and thin malaria smears*

3. Measure hemoglobin.*
4. Collect blood on filter paper for parasite typing.*
5. Collect 50 to 150 µL of blood for antibody*
6. If axillary temperature ≥37.5°C or history of recent fever, or hemoglobin <8.5 g/dL, read blood slides immediately and treat for malaria if parasite positive.

* By fingerprick if not previously collected as part of the vaccine follow up
** Study may terminate prior to Week 24 depending on starting date

7.6.3 Disease Follow Up

Following the first vaccination, clinical trial staff will be available daily in each village. Parents of subjects will be strongly encouraged to bring their child to the clinic if there is any sign of illness. At these unscheduled visits the following will be done:

1. Perform basic history and physical exam (including temperature), emphasizing history of recent febrile episodes.
2. If axillary temperature ≥37.5°C or history of recent fever
    a. Collect blood sample by fingerprick for hemoglobin, malaria thin and thick films, filter paper collection and anti-AMA1 antibody ELISA.
    b. Immediately stain and read blood films.
    c. If blood film is positive, begin malaria treatment.
3. Any other test and treatment as justified by signs and symptoms.

7.7 Treatment for Malaria

Malaria cases will be treated according to the Mali National Malaria Control Program Guidelines.

7.8 Laboratory Testing

Using standard techniques, the MRTC Clinical Laboratories of the DEAP/FMPOS will perform the following tests at the MRTC/DEAP/FMPOS laboratories.

1. Complete blood count plus partial white blood cell differential (granulocyte count, lymphocyte count, and mononuclear cell count)
2. Serum creatinine
3. Alanine aminotransferase (ALT)
4. HBsAg ELISA
5. HCV ELISA
6. Reading of malaria blood films

Anti-AMA1 ELISAs will be done at the MRTC in Bamako, Mali using protocols and standards developed by the MVDB. Results will be entered into the database only after a QA audit by MVDB.

7.9 Immunologic Testing

7.9.1 Antibody Assay

Antibody levels to the AMA1 antigens will be measured in serum by ELISA. Duplicate assays will be done for both 3D7 and FVO. Briefly, microwell plates are coated with antigen solution. Plates are washed with TRIS-buffered saline (TBS) containing Tween-20 (T-TBS) and blocked with TBS containing skim milk powder. After washing with T-TBS, diluted serum samples are added in triplicate and incubated at room temperature. After incubation, unbound antibodies are removed by washing the plates with T-TBS, and alkaline phosphatase-conjugated goat anti-human IgG solution is added to each well and incubated for 2 hours at room temperature. Plates are then washed with T-TBS, followed by adding phosphatase substrate solution to each well; the plates are then covered and incubated for 20 minutes at room temperature for color development. The plates are read immediately at 405 nm with a microplate reader. The optical density values are used to determine antibody concentration for AMA1 by comparing to a standard curve formulated with known positive control sera included on each ELISA plate.

7.9.2 Plasmodium **falciparum** *Genotyping*

Blood from study participants' finger pricks will be spotted onto filter papers at regular intervals throughout the study (see **Appendices B and C**). These specimens will be stored for future *P. falciparum* DNA extraction and sequencing of parasite gene polymorphisms. These stored samples will <u>NOT</u> be used to assess the efficacy of the AMA1-C1/Alhydrogel® vaccine. Rather, the information obtained from the molecular analysis of *P. falciparum* infections in immunized individuals could prove invaluable in designing future malaria vaccines, should the AMA1-C1/Alhydrogel® vaccine prove less than 100% effective.

7.9.3 Growth Inhibition Assay

The Growth Inhibition Assay (GIA) is designed to determine whether anti-AMA1 antibodies obtained from an immunized animal or person can inhibit the process of merozoite invasion into red cells. In this assay, synchronized blood-stage parasites are incubated with sera from volunteers for a period of 40 hours *in vitro*. During this period, merozoites emerge from the infected red cells, invade normal red cells, and initiate a new growth cycle. Parasite growth and development in the newly invaded red cells are assessed in our studies by measuring the activity of a parasite metabolic enzyme—lactate dehydrogenase. Enzyme activity determined by a colorimetric assay is proportional to the number of parasites. Results with immune sera are compared to results with normal nonimmune sera and then expressed as percent inhibition of parasites.

8.0 ADVERSE EVENTS MONITORING AND REPORTING

8.1 Definitions

8.1.1 Adverse Event

An adverse event (AE) includes any noxious, pathological or unintended change in anatomical, physiological or metabolic functions as indicated by physical signs, symptoms and/or laboratory-detected changes occurring in any phase of the clinical study, whether associated with the study vaccine or active comparator, and whether or not considered vaccination related. This includes an exacerbation of pre-existing conditions and intercurrent illnesses. All AEs must be graded for intensity and relationship to the investigational vaccine as described in **Section 8.2.2** and **Section 8.2.3** in this protocol.

8.1.2 Serious Adverse Event (SAE)

An SAE is an AE, whether considered related to the investigational vaccine or not, meeting one of the following conditions:

1. <u>Death</u> during the period of protocol-defined surveillance
2. <u>Life threatening</u>: defined as an event that places a subject at immediate risk of death at the time of the event and does not refer to an event that hypothetically might have caused death were it more severe
3. <u>Hospitalization</u> during the period of protocol-defined surveillance: defined as at least an overnight stay in the hospital or emergency ward for treatment that would have been inappropriate if administered in the outpatient setting
4. Results in a <u>congenital anomaly or birth defect</u>
5. Results in a persistent or significant <u>disability or incapacity:</u> defined as a substantial disruption of the study participant's ability to carry out normal life functions
6. Any other <u>important medical event</u> that may not result in death, be life threatening, or require hospitalization, may be considered a serious AE when, based upon appropriate medical judgment, the event may jeopardize the subject and may require medical or surgical intervention to prevent one of the outcomes listed above.

8.2 Assessment of Adverse Events

8.2.1 Identification of AEs

Assessment of safety will include clinical observation and monitoring of hematological, chemical, and immunologic parameters. Safety will be evaluated by monitoring of volunteers for local and systemic adverse reactions during the course of the trial. Volunteers will be closely monitored for 30 minutes following each immunization. Additionally, volunteers will return to the clinic on Days 1, 2, 3, 7, and 14 following each vaccination for clinical assessments.

All AEs will be graded for intensity and relationship to study product. Reactions will be graded as described in **Section 8.2.2** in this protocol and in Appendices D and E. A study clinician will be available 24 hours a day during the study evaluation period; a study clinician will stay in the study site for the duration of the trial and will be available to the

study participants at all times. Should a volunteer call on a study clinician to report an adverse event, it will be fully documented in the volunteer's study chart, and discussed with the Principal Investigator.

8.2.2 Determination of Severity

All AEs will be assessed by the investigator using the following protocol-defined grading system:

Grade 0 (None)	
Grade 1 (Mild):	No effect on activities of daily living. No intervention required.
Grade 2 (Moderate):	Partial limitation in activities of daily living (can complete ≥ 50% of baseline), or minimal intervention required.
Grade 3 (Severe):	Activities of daily living limited to < 50% of baseline, or medical evaluation with intervention required
Grade 4:	Extreme limitation in activity, significant assistance required; significant medical intervention or therapy required. **All Grade 4 events will be treated as Serious Adverse Events (SAEs).** (see **Section 8.1.2**)

Intensity of the following AEs will be assessed by the investigator as described in **Appendix D**. All laboratory AEs will be graded in severity following the toxicity table in **Appendix E**.

8.2.3 Association with Receipt of the Study Vaccine

All AEs will have their possible relationship to study vaccine assessed using the following terms:

<u>Definite</u>:	Clear-cut temporal association, and no other possible cause.
<u>Probable</u>:	Clear-cut temporal association and a potential alternative etiology is not apparent.
<u>Possible</u>:	Less clear temporal association; other etiologies also possible.
<u>Remote</u>:	Temporal association between the AE and the vaccine or the nature of the event is such that the vaccine is <u>not</u> likely to have had any reasonable association with the observed illness/event (cause and effect relationship improbable but not impossible).
<u>Not Related</u>:	The AE is completely independent of vaccine administration; and/or evidence exists that the event is definitely related to another etiology.

The degree of certainty with which an AE can be attributed to administration of the study vaccine will be determined by how well the event can be understood in terms of one or more of the following:

1. The event being temporally related with vaccination or reproduced on revaccination.
2. A reaction of similar nature having previously been observed with this type of vaccine and/or formulation.

3. The event having often been reported in the literature for similar types of vaccines.

All local (injection-site) reactions will be considered causally related to vaccination.

8.2.4 Clinical Malaria

Clinical malaria is defined as the presence of any parasitemia determined by microscopy accompanied by at least one of the following:

- Axillary temperature ≥37.5°C
- History of fever in the past 7 days
- Hemoglobin less than 8.5 g/dL.

Clinical malaria will be scored as an expected and vaccine unrelated, adverse event. However the frequency of clinical malaria episodes in the test and comparator groups will be enumerated at the 4-week parasitology follow up and end of the parasitology follow up for two reasons:

1. This is the first time that this vaccine has been tested in a sufficiently large target group to determine an impact on clinical malaria. In rare cases, other experimental vaccines have exacerbated the disease they target and the possibility of this occurring for the AMA1-C1/Alhydrogel® vaccine cannot be ruled out. Therefore the frequency and severity of clinical malaria cases in test and control groups will be assessed at the 4-week parasitological follow up and at the end of the parasitological follow up as a safety indicator. Should there be clear evidence that the vaccine exacerbates clinical malaria (e.g. an increased frequency of severe anemia or cerebral malaria), appropriate steps will be undertaken to safeguard children experiencing malaria. This may include unblinding the study.
2. The vaccine may reduce the frequency of clinical malaria attacks. This is not an endpoint of the trial as the intended use of the vaccine is to reduce severe malaria and malaria associated mortality. Reduction in severe malaria or mortality may occur without reduction in the frequency of mild clinical malaria attacks associated with low-grade parasitemia and this trial has not been powered to detect a reduction in relatively mild clinical malaria. Therefore, a lack of reduction in clinical malaria cases should not be construed as a failure of the vaccine. However, should a significant reduction occur, this would be of interest and will be noted.

8.3 Adverse Event Reporting

8.3.1 Serious Adverse Event Reporting

All SAEs will be reviewed by a study physician, recorded on the appropriate SAE form, and followed through to resolution by a study physician. All SAEs will be reported by email, telephone or fax within 1 working day of notification of the SAE occurrence to the PI, to all of the following:

- Sponsor (Regulatory Compliance and Human Subjects Protection Branch [RCHSPB]/NIAID): Phone: 301-846-5301, Fax: 301-846-6224
- FMPOS Institutional Review Board (IRB): Phone: (223) 222-5277

- NIAID Institutional Review Board (IRB): Phone: 301-435-9273, Fax: 301-435-6739
- Medical Monitor: Dr Tatiana Keita, Clinique Pasteur, Bamako, Mali. Phone: +223 229 1010
- Adverse events which meet the criteria for placing the study on hold (as described in Section 8.5) will be reported to the NIAID DSMB Executive Secretary as soon as they occur: Phone: 301 846-6553, FAX 301 846-6224

Following notification from the investigator, RCHSPB as the Investigational New Drug (IND) sponsor, will report events that are both serious and unexpected that are possibly, probably, or definitely related to the vaccine, to the FDA within the required timelines: fatal and life-threatening events within 7 calendar days (by phone or fax) and all other SAEs in writing within 15 calendar days. All SAEs not listed as possibly, probably, or definitely related will be reported to the FDA at least annually in a summary format.

8.3.2 Other Adverse Event Reporting

All local and systemic reactions not meeting the criteria for Serious Adverse Events will be captured on the appropriate case report form (CRF). These events will be followed to resolution. Grade 3 adverse events deemed definitely or probably related to vaccination will be reported by email or fax within 15 working days of the PI becoming aware of the event, to the Sponsor, the NIAID IRB, the FMPOS IRB, and the NIAID DSMB.

8.4 Adverse Event Monitoring

8.4.1 Local Medical Monitor

An independent medical monitor, Dr. Tatiana Keita, has been appointed for oversight of participant safety in this trial. Dr. Keita is a Pediatrician and is independent of Mali Malaria Vaccine Development Unit, the Malaria Research and Training Center, and MVDB. The Medical Monitor will be available to advise the investigators on trial-related medical questions or problems. Should Dr. Keita not be available, Dr. Mariam Sylla or Dr. Fatoumata Dicko will serve as a substitute independent Medical Monitor.

The Medical Monitor's primary responsibility will be to monitor participant safety. The Principal Investigator is responsible for ensuring that the Medical Monitor is aware of any new safety information that becomes available to him during the course of the trial. The Medical Monitor will have access to a copy of the randomization code and can refer to it for safety reasons.

8.4.2 Data and Safety Monitoring Board (DSMB)

The DSMB is a standing committee of NIAID with the local medical monitor as an ad hoc member to advise the study investigators and RCHSPB on the trial. The DSMB's primary responsibility will be to monitor volunteer safety. The DSMB will periodically review individual and cumulative volunteer data on safety and enrollment when making recommendations to put the study on hold (**Section 8.5**) or recommendations for continuation of the study (**Section 8.6**). The code for the vaccine assignments will be sent in a sealed envelope by express mail to the Executive Secretary of the DSMB Regulatory Compliance and Human Subjections Protection, SAIC Frederick, Inc., 5705

Industry Lane, Suite J, Room 208, Frederick, MD 21702 USA; Phone: 301-846-6553/Fax: 301-846-6224; prior to the first vaccination of each cohort.

MVDB will request that the DSMB chair will invite a Malian physician to be an ad hoc member of the DSMB. This physician will be suggested by the senior investigators in Mali and will be independent from the MVDB and the MMVDU.

8.5 Criteria for Placing the Study on Hold

The following criteria will be used as rules to put the study on hold until reviewed by the Medical Monitor, DSMB, the study sponsor (RCHSPB) and the FMPOS and NIAID IRBs:

1. One or more volunteers experience a serious adverse event (SAE) (as defined in **Section 8.1.2** in this protocol) that is determined to be possibly, probably, or definitely related to the vaccine (as defined in **Section 8.2.3** in this protocol), **OR**
2. A volunteer experiences anaphylaxis that is probably or definitely related to the vaccine, **OR**
3. Two or more volunteers in Cohort 1 or 10 % in Cohort 2 alone or 10% of cohorts 2 and 3 combined experience an objective physical finding or laboratory abnormality of Grade 3 (with the exception of isolated Grade 3 erythema or swelling), as defined in **Section 8.2.2** in this protocol, that is determined to be probably or definitely related to the vaccine.

8.6 Approval to Continue the Study

Table 2 Summary of Safety Review Meetings and Vaccination Schedule

Center and (Weeks)	Cohort 1		Cohort 2	Cohort 3
	Group 1	Group2		
Don0	12 A + 6 C			
Don2		12 B + 6 C		
Don4	12A + 6 C			
Don6		12 B + 6 C		
Don9	DSMB and IRBs Safety Review			
Ban0			30 B+ 30 C	
Ban4			30 B+ 30 C	
Ban7	DSMB and IRBs Safety Review			
Ban8				120 B+ 120 C
Ban12				120 B+ 120 C
Don: Donéguébougou, Mali<br>Ban: Bancoumana, Mali<br>A: 20µg AMA1-C1/Alhydrogel®<br>B: 80µg AMA1-C1/Alhydrogel®<br>C: Comparator vaccine (Hiberix™)				

It is the Principal Investigator's responsibility to provide the safety data to the Medical Monitor and the DSMB as specified in **Table 2**. It is the PI's responsibility not to proceed without the approval of the Medical Monitor within a cohort and the approval of the DSMB and IRBs between cohorts as specified in **Table 2** and below. The study cannot proceed without this approval. Occurrence of an SAE will follow the holding rules in **Section 8.5** and the reporting rules in **Section 8.3**. Additionally, any new information that may adversely affect the safety of the subjects or the conduct of the study will be submitted to the Medical Monitor and the DSMB as it becomes available.

The medical monitor will review the safety data one week after each vaccination in Cohort 1 (four times), one week after each vaccination in Cohort 2 (2 times), and one week after each vaccination in Cohort 3. If at any time there is a concern as defined under the holding rules (**Section 8.5**), the study will be put on hold, the safety data will be reviewed by the DSMB, and a decision reached as to the continuation, put on hold or stopping the study. All reports of such DSMB meetings will be submitted to the NIAID

and the FMPOS IRBs. The study cannot move to the next cohort without the approval of the IRBs after reviewing the safety data and the recommendations of the DSMB.

All cumulative safety data reports from the trial for each cohort will be submitted to the DSMB, and the DSMB report will be submitted to both IRBs before the next cohort is vaccinated as follows:

1. The DSMB will review the safety data one week after the last vaccination for Cohort 1 (Group 1: 20 µg of AMA1 on Alhydrogel® or the comparator; d Group 2: 80 µg of AMA1 on Alhydrogel® or the comparator). If there are no concerns or need to put the study on hold (see **Section 8.5**), the DSMB will send a report the NIAID and the FMPOS IRBs. The IRBs, after reviewing the DSMB report and the safety data, will determine if the study can continue to Cohort 2.

2. The DSMB will review the safety data on Cohort 2 one week after the second vaccination with 80 µg of AMA1-C1 on Alhydrogel® or the comparator. The DSMB will also receive any additional safety data from Cohort 1. If there are no concerns or need to put the study on hold (see **Section 8.5**), the DSMB will send a report to the NIAID and the FMPOS IRBs. The IRBs, after reviewing the DSMB report and the safety data, will determine if the study can continue to Cohort 3.

3. As SAEs or grade 3 AEs may occur at any time during the study after vaccination is completed (i.e., during the malaria transmission season), the medical monitor may call for a review of the data at any time because of safety concerns. In addition, the study will be reviewed by the DSMB at the end of September for any evidence of exceptional malaria related complications in the vaccine group.

4. Written approval (via letter or email) to proceed to the next cohort must be obtained from the DSMB and the IRBs prior to vaccination of the next cohort.

5. A final safety and efficacy report will be submitted to the DSMB and IRBs.

It is the Principal Investigators' (or designated agent) responsibility to ensure that the DSMB reviews the current safety data (grouped by cohort), study protocol, and any other requested documents at its meetings. Occurrence of an SAE will be reported to the DSMB at the same time that it is reported to the IRBs. Additionally, any new information that may adversely affect the safety of the subjects or the conduct of the study will be submitted to the DSMB as it becomes available.

9.0 DATA COLLECTION AND MONITORING

9.1 Source Documentation

CRFs will be used to record data for subjects enrolled in the study. In addition, supplementary documents (laboratory test reports, supplementary hospital or medical records, etc.) may form part of the source documentation for a study participant. The Investigator is responsible for the accuracy and completeness of the data reported in the CRFs and other source documents. Data reported in the CRFs that are derived from

source documents should be consistent with source documents and any discrepancies should be explained.

9.2 Study Documentation

Study-related documentation will be completed as required by the IRBs, the sponsor, and regulatory authorities. Continuing review documentation will be submitted by the Investigator to the IRBs on the anniversary date of initial review as specified by each IRB. An annual report will be submitted by the sponsor to the FDA on the anniversary date that the IND for AMA1-C1/Alhydrogel® malaria vaccine went into effect. These reports will provide a brief description of the progress of the investigation as outlined in 21 *Code of Federal Regulations* 312.33, and will include any revisions of the protocol.

The investigators will maintain adequate records of the disposition of the investigational product, including dates, quantity, and use by subjects. If the study is terminated, suspended, or completed, the investigators will destroy or return all unused supplies of the investigational product as specified by the sponsor.

9.2.1 Study Reports

Three unblinded interim safety reports will be prepared by the study statistician Dr. Michael Fay, or his delegate, for presentation to the DSMB. These will include the following data:

1. Cohort 1 to Day 35 of group 2 (*i.e.*, to 7 days post second vaccination)
2. Cohort 2 to Day 35 (*i.e.*, to 7 days post second vaccination)
3. Cohorts 1, 2 and 3 to Week 4 in parasitological follow up (Day 70 for Cohort 3)

The first two reports will include:

1. Line listings of the adverse events for each subject sorted according to dose (Cohort 1 only) and test or control group.
2. Summary of the frequency of immediate, local, and systemic adverse events categorized by severity and by relationship to vaccine in the test and control groups. Where appropriate, the statistical significance of differences in frequencies between test and control groups will be ascertained.

The third report will include the above information to Week 4 of the parasitological follow up and also a summary of the frequency of clinical malaria in the test and control group with geometric mean and range of parasitemia. The mean and range of hemoglobin concentrations with tests to determine if statistical differences occur between test and control groups also will be included.

These reports will not be made available to investigators unless the DSMB advises that the frequency of AEs in the test groups is unacceptable and that the IRBs have not approved continuation of the study.

A final report will be submitted by the investigators to the sponsor after trial completion. This final report will, therefore, contain the safety and immunogenicity data obtained after the follow-up period through to the end of the 2006 malaria transmission season.

9.3 Retention of Records

Trial-related documents will be maintained for a period of 2 years after final marketing approval of the vaccine, or 2 years after the formal discontinuation of clinical development of the product per 21 CFR 312.57 and 21 CFR 312.62. Individual Institutional Review Boards (IRBs) and local authorities may have different requirements for record retention. The site investigator must be aware of all requirements and retain protocol records in accordance with the longest requirement that pertains to the study. No study document should be destroyed without prior written agreement between the RCHSPB, the Principal Investigator and the site investigator. Storage of all trial-related documents will be such that confidentiality will be strictly maintained. Should the site investigator wish to assign the study records to another party or move them to another location, RCHSPB must be notified in writing of the new responsible person and/or the new location.

9.4 Protocol Revisions

No revisions to this protocol will be permitted without documented approval from both the sponsor and the IRBs that granted the original approval for the study. This does not apply to changes made to reduce discomfort or avert risk to study volunteers. Furthermore, in the event of a medical emergency, the investigators shall perform any medical procedures that are deemed medically appropriate. The PI must notify the sponsor of all such occurrences. Any change to the protocol will be submitted to the participating IRBs (NIAID and FMPOS) as a protocol amendment, and changes not affecting risk to volunteers may be expedited, as appropriate.

9.5 Clinical Investigator's Brochure

Investigators will receive the current version of the Clinical Investigator's Brochure, which comprehensively describes all the available preclinical and human experience with the experimental vaccine. If relevant new information becomes available during the course of the trial, the investigators will receive a revised Investigator's Brochure or an amendment to the current version.

9.6 Study Monitoring

The sponsor (RCHSPB or its designee), in collaboration with the World Health Organization (WHO), will monitor all aspects of the study, with respect to current Good Clinical Practice, for compliance with the IRB approved protocol as well as all applicable government regulations. Prior to the start of the study, the PI will be informed of the frequency of monitoring visits and will be given reasonable notification prior to each visit. The objectives of a monitoring visit will be to verify the prompt reporting of SAEs, to check the availability of the signed Informed Consent for enrolled study participants, and to compare CRFs and spreadsheets with other source data for completeness and accuracy. During the monitoring visit, the PI (and/or designee) and other study personnel should be available to discuss the study. Study documents must be available for review throughout the course of the study. The sponsor will retain originals of the Form FDA 1572 and copies of other study documents as deemed necessary.

10.0 STATISTICAL CONSIDERATIONS

10.1 General Design

The goal of this Phase 1/Phase 2 vaccine study is to assess the safety and impact on parasitemia of AMA1-C1/Alhydrogel® malaria vaccine in human volunteers.

10.1.1 Description of the Statistical Methods to be Employed

The purpose of this trial is to estimate adverse event rates and patterns of immune response as well as to compare these rates and patterns between the investigational and comparator vaccines, in different doses of the study vaccine, and to compare the response by serotype.

This section briefly describes the statistical methods to be used; a detailed analytic plan will fully describe the methods. The analytic plan will discuss the planned approaches to missing data. Listings will show all observed data and, if applicable, imputed values and the approaches taken for imputation. Deviations from the original analytic plan will be thoroughly documented and reported to the sponsor.

Estimates will be presented with their 90% confidence intervals. Descriptive approaches will be used to meet most of the objectives of the protocol stated in **Section 2.0**. Formal statistical tests will be used to compare the frequencies of episodes of malaria with a parasitemia >3000 per µL (Cohort 2 and 3, primary objective 2) and to compare the antibody levels by dose and serotype (secondary objective 1). Results will be presented in tables and graphs.

Most of the analyses of immunogenicity will be based on a longitudinal mixed model with terms for dose group and serotype. The detailed analysis plan will describe the method of modeling and the approach to selecting a covariance structure.

Primary Objective 1: To estimate the frequency of vaccine-related AEs, graded by severity, for each dose in Cohort 1 and for all subjects in Cohorts 2 and 3 combined.

a. The frequency of immediate, systemic, and local AEs will be summarized.
b. A line listing of each clinical and laboratory AE classified as immediate (within the first 30 minutes), systemic, and local will be displayed in tables stratified by vaccine allocation and dose cohort.
c. Episodes of clinical malaria will be counted as AEs and the frequency compared between groups by transmission season.
d. AEs will be summarized by severity and relationship to vaccine by individuals and dose cohort.

The proportion of volunteers with at least one local adverse event will be compared by dose cohort, and tests performed to assess whether the groups differ with respect to these proportions.

Primary Objective 2: To determine the decrease in the frequency of parasitic episodes with a parasitemia >3000 per μL in subjects vaccinated with AMA1-C1 compared to subjects vaccinated with comparator (Cohorts 2 and 3 only).

The number of episodes of parasitemia >3000 per μL per subject per day of observation during the transmission season will be assessed for both the test and control subjects in Cohorts 2 and 3 combined. The number of days of observation for each subject will be the time from the first day of parasitological follow up (**Section 7.6.2**) to the last day of the parasitological follow up (**Section 7.6.2**), or the day the subject was withdrawn from the study, which ever is earlier, less a period of 4 weeks for each time the subject was treated for malaria. Parasitemic episodes occurring during the 4-week post-treatment period will not be counted. A Wilcoxon-Mann-Whitney test will be used to test for significant differences in the number of episodes of parasitemia >3000 per μL per subject per day between the AMA1-C1 group and the comparator group (Cohorts 2 and 3 only).

Secondary Objective 1: To describe the kinetics of the antibody response to AMA1-FVO and AMA1-3D7 following vaccination and during subsequent infection, as measured by enzyme-linked immunosorbent assay (ELISA).

Anti-AMA1 antibody will be measured by ELISA using AMA1 3D7 and AMA1 FVO serotypes on vaccine study Days 0, 14, 28, 42, 98 154, 210, 294 and 364. To exploit the multiple measures of antibody within each subject, a longitudinal model that accounts for the covariance both across time and between serotypes will be built to describe the antibody response over time and by different serotypes. The model will include all three groups (Comparator, AMA1-C1 20 μg, and AMA1-C1 80 μg) for Cohort 1 or two groups (Comparator and AMA1-C1) for Cohorts 2 and 3 and terms for time and the AMA1 FVO or AMA1 3D7 serotype. The level of antibody at Day 42 will be estimated from the resulting model collapsing over the two serotypes. Formal statistical tests will assess whether the response is monotone (comparator vaccine, AMA1-C1 20 μg, and AMA1-C1 80 μg) for the three groups.

Various exploratory methods will be used to assess the sensitivity of the results to the assumptions in the model.

For subjects who had one or more observed malaria infections during the parasitological follow up, further ELISAs will be done to measure the change in antibody levels during the acute and convalescent phase.

Secondary Objective 2: To describe the AMA1 genotype of parasite before and after vaccination with AMA1-C1/Alhydrogel® or with comparator vaccine.

An attempt will be made to amplify the AMA1 gene from the DNA in each filter paper sample with a parasitemia > 3000 per μL and a random sample of 20% of all other filter paper samples. If the amplification is successful, the hypervariable segment of region I of the AMA1 gene will be sequenced. Mixed genotypes are expected in a proportion of the samples amplified. Where possible, these will be deconvoluted. A subset of the samples will have the AMA1 gene fully sequenced. If these longer sequences show

evidence of recombination, additional regions outside the hypervariable region I segment may be sequenced for all samples with hypervariable genotypes corresponding to the recombinant types.

Descriptive statistics will be used to list genotypes of samples classified by village (Donéguébougou or Bancoumana), by vaccinated or comparator groups, and by the level of parasitemia.

Secondary Objective 3: To examine the use of other parasitological and disease measures of vaccine effect as the basis for future Phase 2 malaria vaccine design.

Secondary Objective 4: To measure the inhibition of parasite growth as measured by the in vitro GIA to both FVO and 3D7 parasite clones.

Graphs will display growth inhibition expressed as a percent of inhibition comparing test sera to preimmune sera. Depending on the distribution of the data, parametric or non-parametric methods will be used to compare inhibition as a function of dose, and clone.

Secondary Objective 5: To determine the relationship between anti-AMA1 antibody concentration, as judged by ELISA, and degree of in vitro growth inhibition of *P. falciparum* in a GIA.

10.1.2 Safety

The primary safety endpoint is the frequency of vaccine-related AEs, as classified by both intensity and severity through active and passive surveillance. Separate assessments of systemic and local reactions will be performed. Comparisons will be made between the AMA1-C1/Alhydrogel® and the comparator vaccine.

10.1.3 Immunogenicity Analysis

Anti-AMA1 antibody will be measured by ELISA on Days 0, 14, 28, 42, 98, 154, 210 294, and 364 as listed in the schedule of visits (see **Appendix B** in this protocol) and other selected samples following evidence of infection.

10.1.4 Premature Termination

Should the study be terminated early, the investigative team will determine which study questions can be addressed in an unbiased manner with the available data. The available data will be analyzed and interpreted in light of early termination.

10.2 Sample Size

Sample sizes in this study have been determined by safety considerations alone (Cohort 1) or on the ability to detect an effect of the vaccine on parasitological outcomes (combined size of Cohort 2 and Cohort 3) during a minimum follow up through September, October, November, and December with vaccinations competed in August. Should the vaccinations be completed earlier, follow up will start earlier on Cohort 3 day 42 but extend until the planned date. This would result in a small increase in power.

The relative proportion in Cohort 2 and Cohort 3 was decided on safety considerations as detailed below.

Cohort 1:
In previous studies, there have been 35 adults vaccinated three times with 80 µg of AMA1-C1/Alhydrogel® on a 0, 1, 2 month, a 0, 1, 6 month, or a 0, 1, 12 month schedule. A further 43 adults have been vaccinated with either 5 or 20 µg in a 3-shot schedule. There have been no substantial safety issues with these adult vaccinations. Therefore, we believe that there is ample safety data to proceed with a two shot vaccination (one less than in adults) using 20 µg of AMA1-C1/Alhydrogel® in 12 children as the first group of a dose and number escalation Phase 1/Phase 2 program in children, especially as most registered childhood vaccines use the same dose as an adult. These 12 children receiving 20 µg of AMA1-C1/Alhydrogel® will provide sufficient safety data for immunizing a further group of 12 children with the anticipated maximum dose of two shots of 80 µg of AMA1-C1/Alhydrogel®. These two groups of 12 in the Phase 1 study provide the baseline safety data for the subsequent Phase 2 study.

Cohort 2: As detailed below, 150 children are required to provide sufficient power for the Phase 2 study. To reduce the risk of multiple grade 3 AEs or SAEs should the vaccine cause unexpected reactogenicity, 30 children (Cohort 2) in the Phase 2 will be vaccinated first and observed for AEs before the remainder are vaccinated. The approximately two-fold increase in numbers (from 12 at the chosen dose in Cohort 1 to the 30 in Cohort 2) represents a very conservative increase in total numbers vaccinated. Following vaccination of Cohort 2, a safety review will take place of all 42 subjects at the chosen dose of Cohort 1 and Cohort 2 prior to vaccinating the 120 subjects in Cohort 3 with the malaria test vaccine. Thus, there will be less than a three-fold escalation in the number of children vaccinated in Cohort 3. This represents a conservative increase that makes it unlikely that multiple grade 3 AEs or SAEs will occur in Cohort 3.

Cohort 2 and 3: Both contribute to a group of 150 for measuring a biological effect of vaccination on parasitemia.

Longitudinal studies in Donéguébougou in 1999 and 2000 examined many potential end points for Phase 2 studies. These included parasitogical, hematological and disease outcomes. From these, the number of episodes of malaria with a parasitemia of greater than 3000 per µL has been chosen as the primary outcome for this Phase 2. The reasons include the precision of the case definition, the correlation between this level of parasitemia and frequency of uncomplicated malaria, and ease of measure and the biological relevance in predicting whether a vaccine may achieve a reduction in severe malaria. In particular, there is considerable evidence that reduction in the number of high parasitemic episodes is likely to impact on the probability of a child experiencing severe malaria. Although the detailed case definition is different, this choice of endpoint is in general agreement with reduction in average parasite density used as the endpoint of the only Phase 2 vaccine trial conducted to date, to test a recombinant blood stage malaria vaccine [34].

As previously stated, a highly effective blood stage malaria vaccine is not expected to result in sterile immunity. In this area surrounding Bamako that includes Donéguébougou and Bancoumana, children have developed sufficient natural immunity to cease to be at significant risk of severe malaria at approximately 8 years old. However, they still have episodes of malaria with a parasitemia of >3000 per µL, although at a substantially lower rate than in younger children. Therefore, the reduction in frequency of episodes of malaria with a parasitemia >3000 per µL from what is observed in a 3 year old to the frequency seen in an 8 year old has been chosen as the effect expected for a 100% effective vaccine. A rate of 0.05 episodes of parasitemia >3000 per µL per infectious bite was estimated for the 3 year olds, and a rate that was 56% lower was estimated for 8 year olds. This was used as the basis of the power calculation.

Previous studies show considerable heterogeneity in the immune response of individuals and parasites in this area because of substantial diversity in their AMA1 sequences. Therefore, the study has been powered to detect a 56% reduction in the rate of parasitic episodes >3000 per µL per infectious bite in 50% of the vaccinees. The methods described in Fay et al, based on the combined data from 1999 and 2000, were used to calculate group size based on these assumptions. The estimates of the frequency at which parasitic episodes >3000 per µL occur in 3 year olds in Donéguébougou in 1999 and 2000 are limited by relatively small sample sizes. Thus, confidence limits on the estimated group size were calculated using a bootstrap method and the 90% upper confidence limit of 105 subjects in each of the control and test groups chosen to give a power of 80% and $\alpha = 0.05$. This estimated minimum group size, based on observation through a complete transmission season, was increased to 150 per group to allow for follow up commencing part way through the transmission seasons (starting at the end of August) and for drop outs.

11.0 PROTECTION OF HUMAN SUBJECTS

11.1 Institutional Review Board/Ethics Committee

The investigators will be responsible for obtaining IRB approvals for the study. Before the start of the study, the appropriate documents (including the protocol, Investigator's Brochure, and informed consent form) will be submitted to the IRBs. A copy of the study approval (including approval of the informed consent form) is to be maintained in the study document binder and a copy will be supplied to the sponsor. During the study, the PI is responsible for providing the IRBs with all documents subject to review (i.e., protocol amendments, informed consent form updates, and any written information that may be provided to the subject). Annual reports on the progress of the study will be made to the IRBs by the investigators in accordance with IRB guidelines and government regulations.

11.2 Informed Consent

In obtaining and documenting informed consent, the Investigator must comply with the applicable regulatory requirements, current Good Clinical Practice, and ethical principles. The written informed consent form must be approved by all IRBs prior to its use.

11.3 Risks

Risks to the volunteers are associated with venipuncture and with immunization. These risks are outlined below.

11.3.1 Venipuncture

Risks occasionally associated with venipuncture include pain and bruising at the site of venipuncture, hematoma, infection, lightheadedness, and syncope (rarely).

11.3.2 Immunization with AMA1-C1/Alhydrogel®

Possible local vaccine reactions include pain, swelling, erythema, induration, limitation of limb movement for several days, lymphadenopathy, or pruritis at the injection site. Local subcutaneous nodules, believed to be granulomatous reactions to aluminum hydroxide, have been observed with use of aluminum hydroxide-based adjuvants. Thus, most aluminum hydroxide-adsorbed vaccines are injected intramuscularly rather than subcutaneously. Systemic reactions such as fever, chills, headache, fatigue, malaise, myalgia, and joint pain may also occur. Immediate hypersensitivity reactions including urticaria, anaphylaxis, or other IgE-mediated responses are possible as with any vaccine. As with any investigational vaccine, there is a theoretical possibility of risks about which we have no present knowledge. Volunteers will be informed of any such risks should further data become available.

11.3.3 Immunization with Comparator Vaccine

Local adverse reactions are common and include redness, swelling, and pain [33]. Fever, loss of appetite, vomiting, diarrhea, or restlessness may develop in some patients after the injection. Uncommon adverse reactions (< 1%) including injection site mass, fatigue, purpura, emotional lability, rash, and urticaria have been reported. Rarely, anaphylactoid reactions, collapse, and shock-like state have been reported following administration of Hiberix™.

11.4 Precautions Taken to Minimize Risks

11.4.1 Immunization

As outlined above, the participants will be monitored closely during their participation in this study. The study vaccines have been produced according to Good Manufacturing Practice (GMP). The vaccines will be administered by experienced investigators with drugs and equipment available for the treatment of anaphylaxis and other potential adverse reactions. All vaccine doses will be given by intramuscular injection to minimize injection site reactions such as pain.

11.4.2 Protection of Study Staff

All study personnel have been trained to follow Universal Precautions. Additionally, the following approved Standard Operating Procedures from the MRTC clinical lab elaborate the precautions that will be taken by study personnel to minimize risks: General

Laboratory Safety, Exposure to Blood and Infectious Material, and Waste Management. The procedures for handling exposures to blood and infectious material are in keeping with the rules and regulations of the local Malian authorities, including the Malian initiative addressing access to antiretroviral medications.

11.5 Benefits

Volunteers may not receive any direct benefit from participation in this study. It is hoped that information gained in this study will contribute to the development of a safe and effective malaria vaccine. Volunteers who receive the comparator vaccine (Hiberix™) during the course of the study proper are protected against Haemophilus influenzae type b disease. Volunteers who receive the AMA1-C1/Alhydrogel® vaccine during the trial will be offered Hiberix™ after the conclusion of the study, and thus will also receive this benefit. This will be administered at the recommended schedule of a single dose.

Free medical treatment will be provided to all enrolled participants during the active immunization phase and the follow-up period. The pharmacy at the clinic will have sufficient provisions to provide participants with drugs for the treatment of minor illnesses free of charge. If further evaluation or treatment is necessary, the participant will be referred one of the National Hospitals in Bamako or Kati located within 60 km of Donéguébougou and Bancoumana. If the investigators judge that a participant requires hospitalization in Bamako or Kati, referral and transportation to these places will be arranged and the medical management of the participant will be monitored by senior physician-investigators and the local Medical Monitor. Medical care for ailments not related to vaccination will not extend beyond the study period. Medical care for ailments related to vaccination will extend at least until the condition has resolved.

In practice, the MRTC-run clinic in Donéguébougou has been the primary provider of medical care in Donéguébougou since 1995. This clinic provides malaria care free-of-charge; hence, the entire community – and not just study participants – benefits from its presence. Study doctors at the MRTC-run clinic in Donéguébougou will provide malaria care regardless of whether a subject enrolls in the study. In Bancoumana alternative sources of care are available. In both sites medical care is available regardless of study participation.

Study participants who develop clinical malaria during the course of the study will be treated according to the guidelines of the Malian National Malaria Control Program. All medications used for treatment are licensed in Mali and have proven safety records.

11.6 Confidentiality

All study-related information will be stored securely at the study site in Donéguébougou, Bancoumana, or at the MRTC offices in Bamako, Mali. All participant information will be stored in locked file cabinets in areas with access limited to study staff. All laboratory specimens, reports, study data collection, process, and administrative forms will be identified by coded number only to maintain participant confidentiality. All computer entries will be done by coded numbers only, and all local databases will be secured with password-protected access systems. Forms, lists, logbooks, appointment books, and any

other listings that link participant ID numbers to other identifying information will be stored in a separate, locked file in an area with limited access.

Participants' study information will not be released without the written permission of the participant, except as necessary for monitoring by NIAID and/or its contractors, the FDA, and the WHO monitor.

11.7 Compensation

Volunteers will not be given any monetary compensation. Volunteers will be given 75 kg of rice and 75 kg millet, in three equal installments, to compensate for the time taken to come to the study clinic for study-related visits. The amount of cereal has been determined after consultation with village elders, and represents an equivalent for the amount of time taken from working in their fields (total value of approximately USD$90). The first installment will be distributed after <u>all</u> participants of the cohort have received the first injection, the second will be distributed after one month after the second immunization, and the third will be given at the conclusion of the study.

Throughout Mali, the availability of food is subject to seasonal variation in relation to the harvest season. However, there is no recent history of famine or starvation. In the region of our study site, while cases of pediatric malnutrition are occasionally seen at the village health clinic, these are attributable to poor feeding habits rather than to scarcity of food, and the intervention is to educate parents to provide more nutritional foods to small children. The total amount of food to be distributed in three parts over the course of one year will last an average family approximately four weeks. The type of food distributed – rice and millet – are staple starches that are typically served accompanied by a sauce containing some sort of meat as well as vegetables, and therefore, are only a part of the local diet. This amount of compensation is consistent with what we have provided to participants of longitudinal studies in Mali for several years, and has been carefully considered by the local Malian IRB, who has determined that it is appropriate compensation for time lost to study procedures and is not coercive.

Screened subjects not eligible for enrollment will receive medical care and referral as appropriate. Compensation will not be offered to participants who fail screening.

12.0 REFERENCES

1. World Health Organization 2002. Annex Table 3: Burden of disease in DALYs by cause sex and mortality stratum in the WHO regions, estimates for 2001. World Health Report 2002: Reducing risks, Promoting Healthy life. Geneva: World Health Organization 192-197.
2. Duflo B et al 198 . Estimation of the impact of principal diseases in rural Mali. Rev Epidemiol Santé Publique 1986; 34(6):405-18.
3. Programme National de Lutte Contre le Paludisme. Politique Nationale de Lutte Contre le Paludisme au Mali. Deuxième revision. Bamako, Mali (2003).
4. Niambele, M.B. Thèse de Medicine. Université de Bamako, Bamako, Mali (1999).
5. Poudiougou, B., Cisse, M.B., Ranque, S., et al**.** Risk Factors of Severe and Complicated Malaria and Malaria Specific Mortality in Pediatric Hospital in Bamako, Mali. *Am. J. Trop. Med. Hyg.* 69(3) Suppl:305-6 (2003).
6. Kone, A., Dicko, A., Coulibaly, D., et al**.** Clinical Presentation and Risk Factors for Death from Severe Falciparum Malaria in Bandiagara, Mali. *Am. J. Trop. Med. Hyg.* 69(3) Suppl:317-8 (2003).
7. Good, M.F., Kaslow, D.C. & Miller, L.H. Pathways and strategies for developing a malaria blood-stage vaccine. *Annu. Rev. Immunol.* 16, 57-87 (1998).
8. Narum, D.L. & Thomas, A.W. Differential localization of full-length and processed forms of PF83/AMA1 an apical membrane antigen of *Plasmodium falciparum* merozoites. *Mol. Biochem. Parasitol.* 67, 59-68 (1994).
9. Crewther, P.E., Culvenor, J.G., Silva, A., Cooper, J.A. & Anders, R.F. *Plasmodium falciparum*: two antigens of similar size are located in different compartments of the rhoptry. *Exp. Parasitol.* 70, 193-206 (1990).
10. Thomas, A.W., Bannister, L.H., Waters, A.P. Sixty-six kilodalton-related antigens of Plasmodium knowlesi are merozoite surface antigens associated with the apical prominence. Parasite Immunol. 12, 105-13 (1990).
11. Kocken, C.H. et al. Precise timing of expression of a *Plasmodium falciparum*-derived transgene in *Plasmodium berghei* is a critical determinant of subsequent subcellular localization. *J. Biol. Chem.* 273, 15119-15124 (1998).
12. Deans, J.A. et al. Rat monoclonal antibodies which inhibit the in vitro multiplication of *Plasmodium knowlesi*. *Clin. Exp. Immunol.* 49, 297-309 (1982).
13. Thomas, A.W., Deans, J.A., Mitchell, G.H., Alderson, T. & Cohen, S. The Fab fragments of monoclonal IgG to a merozoite surface antigen inhibit *Plasmodium knowlesi* invasion of erythrocytes. *Mol. Biochem. Parasitol.* 13, 187-199 (1984).
14. Waters, A.P. et al. A merozoite receptor protein from *Plasmodium knowlesi* is highly conserved and distributed throughout *Plasmodium. J. Biol. Chem*. 265, 17974-17979 (1990).
15. Marshall, V.M., Zhang, L., Anders, R.F. & Coppel, R.L. Diversity of the vaccine candidate AMA1 of *Plasmodium falciparum*. *Mol. Biochem. Parasitol.* 77, 109-113 (1996).
16. Oliveira, D.A. et al. Genetic conservation of the *Plasmodium falciparum* apical membrane antigen-1 (AMA1). *Mol. Biochem. Parasitol.* 76, 333-336 (1996).
17. Hodder, A.N. et al. The disulfide bond structure of *Plasmodium* apical membrane antigen-1. *J. Biol. Chem.* 271, 29446-29452 (1996).

18. Thomas, A.W., Waters, A.P. & Carr, D. Analysis of variation in PF83, an erythrocytic merozoite vaccine candidate antigen of *Plasmodium falciparum*. *Mol. Biochem. Parasitol.* 42, 285-287 (1990).
19. Escalante, A.A. et al. Polymorphism in the gene encoding the apical membrane antigen-1 (AMA1) of *Plasmodium falciparum*. X. Asembo Bay Cohort Project. *Mol. Biochem. Parasitol.* 113, 279-287 (2001).
20. Dolo, A., Thera, M.A., Baby, M., et al. Strain-specific antibody responses to potential malaria vaccine antigens in Mali. *Am. J. Trop. Med. Hyg.* 69(3) Suppl:495 (2003).
21. Hodder, A.N., Crewther, P.E. & Anders, R.F. Specificity of the protective antibody response to apical membrane antigen 1. *Infect. Immun.* 69, 3286-3294 (2001).
22. Kennedy MC, Wang J, Zhang Y, Miles AP, Chitsaz F, Saul A, et al. In vitro studies with recombinant Plasmodium falciparum apical membrane antigen 1 (AMA1): production and activity of an AMA1 vaccine and generation of a multiallelic response. Infect Immun 2002;70(12):6948-60.
23. Sow SO, Diallo S, Campbell JD, Tapia MD, Keita T, Keita MM, Murray P, Kotloff KL, Levine MM. Burden of invasive disease caused by Haemophilus influenzae type b in Bamako, Mali: impetus for routine infant immunization with conjugate vaccine. Pediatr Infect Dis J. 2005 Jun;24(6):533-7.
24. GlaxoSmithKline, UK. Electronic Medicines Compendium Document last updated May 04, 2004. http://emc.medicines.org.uk/emc/assets/c/html/displaydoc.asp?documentid=4864
25. Dicko A, Klion AD, Thera MA, Sagara I, Yalcouye D, Niambele MB, Sogoba M,Dolo G, Dao A, Diallo DA, Doumbo OK, Miller LH. The etiology of severe anemia in a village and a periurban area in Mali. Blood. 2004 Aug 15;104(4):1198-200. Epub 2004 Apr 27.
26. Toure YT, Doumbo O, Toure A, Bagayoko M, Diallo M, Dolo A, Vernick KD, Keister DB, Muratova O, Kaslow DC. Gametocyte infectivity by direct mosquito feeds in an area of seasonal malaria transmission: implications for Bancoumana, Mali as a transmission-blocking vaccine site. *Am J Trop Med Hyg.* 1998 Sep;59(3):481-6.
27. Dolo A, Camara F, Poudiougo B, Toure A, Kouriba B, Bagayogo M, Sangare D, Diallo M, Bosman A, Modiano D, Toure YT, Doumbo O. [Epidemiology of malaria in a village of Sudanese savannah area in Mali (Bancoumana). 2. Entomo-parasitological and clinical study] [Article in French] *Bull Soc Pathol Exot.* 2003 Nov;96(4):308-12.
28. Genton B, Betuela I, Felgler I, Al-Yaman F, Anders RF, Saul A, Rare L, Baisor M, Lorry K, Brown GV, Pye D, Irving DO, Smith TA, Beck HP, Alpers MP. A recombinant blood-stage malaria vaccine reduces Plasmodium falciparum density and exerts selective pressure on parasite populations in a phase 1-2b trial in Papua New Guinea. J Infect Dis. 2002; 185, 820-7.

Appendix A – Malaria Vaccine Consent Comprehension Exam

MALARIA COMPREHENSION EXAM

Double-blind, randomized, controlled Phase 1 and Phase 2 Study of the Safety and Immunogenicity of AMA1-C1/Alhydrogel® Vaccine for *Plasmodium falciparum* Malaria, in children in Donéguébougou and Bancoumana, Mali

____________________ ______________________________

Census ID # Name (first, last)

1. As part of the study, your child will be injected with a live malaria parasite…...………………………………………………………………….…T F
2. There is a chance your child could get sick from this vaccine……………..…………………………………………………………………T F
3. If you change your mind about your child being in the study after your child is vaccinated, you can withdraw your consent for your child...………………………T F
4. This vaccine has been given to hundreds of people already, so we know it is completely safe…………………………………………..…….………...…T F
5. Your child will have blood drawn as part of this study……………………………T F
6. Your child will get 2 vaccinations in this study……………………………………T F
7. If your child feels sick during the study, you shouldn't tell anyone…………...….T F
8. If your child joins the study, your child will need to be followed in our clinic for 12 months…..…………………………………………………………………...…T F
9. Everybody in this study will get the same kind of vaccine…………………..….…T F

- Total number correct before review…………….……..……… ___
- Total number correct after review…………………………….. ___

Reviewed by ______________________________ Date ___/___/___

Parent's signature _________________________ Date ___/___/___

Witness signature __________________________ Date ___/___/___

Appendix B – Schedule of Visits

**Procedures**	**Blood Volume**	**Day**	Pre	0	1	2	3	7	14	28	29	30	31	35	42	98	154	210	294	364
Complete History/Physical			X																	
Obtain Informed Consent			X																	
Interim Clinical Evaluation				X	X	X	X	X	X	X	X	X	X	X	X	X	X	X	X	X
CBC	2 mL		X	X			X	X	X	X			X	X	X	X	X	X	X	X
Anti-AMA1 antibody ELISA				X					X	X					X	X	X	X	X	X
ALT			X	X			X		X	X			X		X					
Creatinine			X	X			X		X	X			X		X					
HCV ELISA	3 mL		X																	
HBsAg ELISA			X																	
Anti-AMA1 antibody GIA				X											X		X			X
Urinalysis			X																	
**VACCINATION**				**X**						**X**										
Filter paper blood collection				X					X	X					X	X	X	X	X	X
**Blood Volume (mL)**			5	5			5	2	5	5			5	2	5	2	2	2	2	2
**Total Blood Volume (mL)**			5	10			15	17	23	28			33	35	40	42	44	46	48	50

Appendix C – Summary of Parasitological Follow Up

Procedures[a]	Parasitology Week		0	1	2	3	4	5	6	7	8	9	10	11	12	13	14	15	16	17	18	19	20	21	22	23	24
	VaccineWeek[b]		6								14								22								30
Temperature, History and Physical exam			X	X	X	X	X	X	X	X	X	X	X	X	X	X	X	X	X	X	X	X	X	X	X	X	X
Weight and Height			X																								
Malaria Smears[c]			X				X				X				X				X					X			X
Hemoglobin[d]			X				X				X				X				X					X			X
Filter paper blood collection			X				X				X				X				X					X			X
Anti-AMA1 ELISA			X				X				X				X				X					X			X

[a] All blood collections by fingerprick unless collection coincides with blood draw for vaccine follow up.
[b]Vaccine follow up for Cohort 3 only. Cohort 1 and 2 will differ; see Section 7.6.2
[c]Only read malaria blood smear at time of examination if temperature ≥ 37.5°C, there is a history of fever in past week, or hemoglobin < 8.5 g/dL
[d] Hemoglobin measured before subject leaves clinic

Appendix D - Table of Assessment of Adverse Event Intensity

Adverse Event	Grade	Intensity
Pain at injection site	0	Absent
	1	Pain that is easily tolerated
	2	Pain that interferes with daily activity
	3	Pain that prevents daily activity
Erythema at injection site	0	0 mm
	1	>0 - ≤20 mm
	2	>20 - ≤50 mm
	3	>50 mm
Swelling at injection site	0	0 mm
	1	>0 - ≤20 mm
	2	>20 - ≤50 mm
	3	>50 mm
Fever (oral)	0	≤37.5°C
	1	37.6°C - 38.0°C
	2	>38.0°C – 39.0°C
	3	>39.0°C
Headache	0	None
	1	Headache that is easily tolerated
	2	Headache that interferes with daily activity
	3	Headache that prevents daily activity
Nausea	0	None
	1	Nausea that is easily tolerated
	2	Nausea that interferes with daily activity
	3	Nausea that prevents daily activity
Malaise	0	None
	1	Malaise that is easily tolerated
	2	Malaise that interferes with daily activity
	3	Malaise that prevents daily activity
Myalgia	0	None
	1	Myalgia that is easily tolerated
	2	Myalgia that interferes with daily activity
	3	Myalgia that prevents daily activity
Arthralgia	0	None
	1	Joint pain that is easily tolerated
	2	Joint pain that interferes with daily activity
	3	Joint pain that prevents daily activity
Urticaria	0	None
	1	Requiring no medications
	2	Requiring PO or topical treatment or IV medication or steroids for <24 hours
	3	Requiring IV medication or steroids for >24 hours

Appendix E – Toxicity Table for Grading Laboratory Adverse Events

This table is to be used to assess laboratory adverse events for those tests to be performed as part of the AMA1-C1 malaria vaccine clinical trial protocol.

ABBREVIATIONS: Abbreviations utilized in the Table:
ULN = Upper Limit of Normal LLN = Lower Limit of Normal

ESTIMATING SEVERITY GRADE

GRADE 1 **Mild:** no effect on activities of daily living; no medical intervention/therapy required
GRADE 2 **Moderate:** partial limitation in activities of daily living (can complete ≥ 50% of baseline); no or minimal medical intervention/therapy required
GRADE 3 **Severe:** activities of daily living limited to < 50% of baseline; medical evaluation/therapy required
GRADE 4 **Life-threatening:** Extreme limitation in activity, significant assistance required; significant medical intervention or therapy required. All Grade 4 events will be treated as Serious Adverse Events (SAEs).

**HEMATOLOGY**					
	Normal Range	Grade 1	Grade 2	Grade 3	Grade 4 (SAE)
Hemoglobin Male and female (aged 2-4)	8.5 – 11.5 g/dL[a]	7.5 – 8.4 g/dL	6.1 – 7.4 g/dL	5.0 – 6.0 g/dL	< 5.0 g/dL
Platelets	133,000 – 523,000/mm^3 [a]	75,000 - 99,999/mm^3	50,000 - 74,999/mm^3	20,000 - 49,999/mm^3	<20,000/mm^3
WBCs	5,900 – 14,400/mm^3 [a]	14,500 – 16,000/ mm^3	16,001- 18,000 /mm^3	18,001- 30,000/mm^3	>30,000 or <1,000 /mm^3
Absolute Granulocyte Count	1,000 – 6,900/mm^3 [b]	800 -999/mm^3	650-799/mm^3	500-649/mm^3	<500/mm^3
**CHEMISTRIES**					
	Normal Range	Grade 1	Grade 2	Grade 3	Grade 4 (SAE)
Creatinine	0.2–0.8 mg/dL (15.2–61 mmol/L)[c]	1.1 - 1.5 x ULN	1.6 - 3.0 x ULN	3.1 - 6 x ULN	> 6 x ULN or dialysis required
ALT	3.9 – 49.6 U/L[a]	1.25 - 2.5 x ULN	2.6 - 5 x ULN	5.1 - 10 x ULN	> 10 x ULN

**URINALYSIS**				
	Grade 1	Grade 2	Grade 3	Grade 4 (SAE)
Proteinuria	2+ or 500 mg - 1 gm/day	3+ or 1- 2 gm/day	4+or 2-3.5 gm/day	nephrotic syndrome or > 3.5 gm/day
Hematuria	5-10 rbc/hpf or 2+	>10 rbc/hpf or 3+	gross, with or without clots, OR red blood cell casts	requires hospitalization

[a]Determined from normal children in Donéguébougou
[b] From Lugada, E. S. et al. Population-Based Hematologic and Immunologic Reference Values for a Healthy Ugandan Population. *Clin Diag Lab Immunol*, 11, 29-34.
[c] From Current Pediatric Diagnosis & Treatment, 17th edition, 2005.
